# MSstatsTMT: Statistical Detection of Differentially Abundant Proteins in Experiments with Isobaric Labeling and Multiple Mixtures

**DOI:** 10.1074/mcp.RA120.002105

**Published:** 2020-11-25

**Authors:** Ting Huang, Meena Choi, Manuel Tzouros, Sabrina Golling, Nikhil Janak Pandya, Balazs Banfai, Tom Dunkley, Olga Vitek

**Affiliations:** 1 Khoury College of Computer Sciences, Northeastern University, Boston, MA, USA; 2 Roche Pharma Research and Early Development, Pharmaceutical Sciences-BiOmics and Pathology, Roche Innovation Center Basel, Hoffmann-La Roche Ltd, Basel, Switzerland

**Keywords:** Mass spectrometry, statistics, quantification, bioinformatics software, mathematical modeling, hypothesis testing, multiple mixtures, protein quantification, TMT

## Abstract

Tandem mass tag (TMT) is a multiplexing technology widely-used in proteomic research. It enables relative quantification of proteins from multiple biological samples in a single MS run with high efficiency and high throughput. However, experiments often require more biological replicates or conditions than can be accommodated by a single run, and involve multiple TMT mixtures and multiple runs. Such larger-scale experiments combine sources of biological and technical variation in patterns that are complex, unique to TMT-based workflows, and challenging for the downstream statistical analysis. These patterns cannot be adequately characterized by statistical methods designed for other technologies, such as label-free proteomics or transcriptomics. This manuscript proposes a general statistical approach for relative protein quantification in MS- based experiments with TMT labeling. It is applicable to experiments with multiple conditions, multiple biological replicate runs and multiple technical replicate runs, and unbalanced designs. It is based on a flexible family of linear mixed-effects models that handle complex patterns of technical artifacts and missing values. The approach is implemented in *MSstatsTMT*, a freely available open-source R/Bioconductor package compatible with data processing tools such as Proteome Discoverer, MaxQuant, OpenMS, and SpectroMine. Evaluation on a controlled mixture, simulated datasets, and three biological investigations with diverse designs demonstrated that *MSstatsTMT* balanced the sensitivity and the specificity of detecting differentially abundant proteins, in large-scale experiments with multiple biological mixtures.

## Teaser

MSstatsTMT implements a general statistical approach for relative protein quantification and tests for differential abundance in mass spectrometry-based experiments with TMT labeling. It is applicable to experiments with multiple conditions, multiple biological replicate runs and multiple technical replicate runs, and unbalanced designs. Evaluation on a controlled mixture, simulated datasets, and three biological investigations with diverse designs demonstrated that MSstatsTMT balanced the sensitivity and the specificity of detecting differentially abundant proteins, in large-scale experiments with multiple biological mixtures.

## Graphical Abstract

**Figure:**
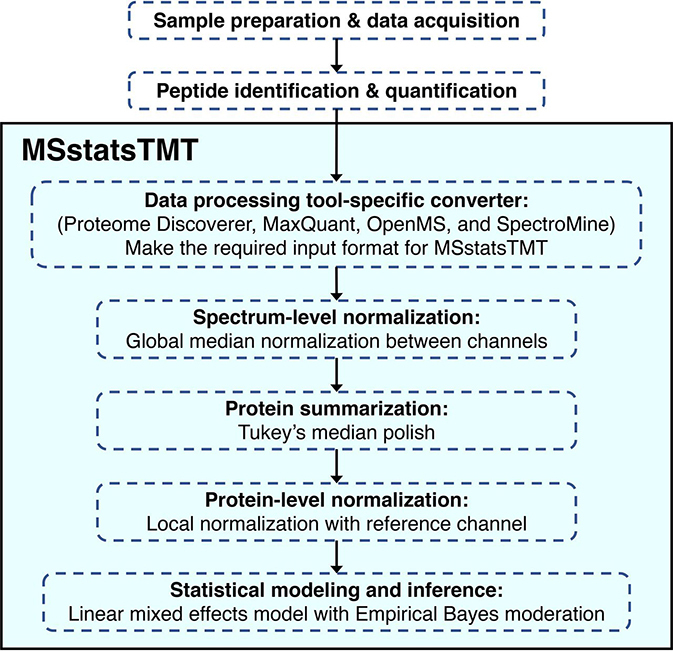

**Highlights**

• Statistical approach for differential abundance analysis for proteomic experiments with TMT labeling.
• Applicable to large-scale experiments with complex or unbalanced design.
• An open-source R/Bioconductor package compatible with popular data processing tools.

Isobaric labeling of biological samples, combined with shotgun MS, is a popular strategy for quantitative proteomics (1, 2, 3). Two most used isobaric labeling methods are Tandem Mass Tags (TMT) (4) and isobaric Tags for Relative and Absolute Quantitation (iTRAQ) (5). In these experiments, peptides from different samples are labeled with isobaric variants of a mass tag (also called channels) and combined to produce a single biological mixture. Commercial TMT reagents now routinely combine up to 16 samples in a mixture (6) and lately new 16-plex reagents (TMTpro) have been released. Because all the variants of the reagent tag have equal molecular mass, the peptides from all the samples form a single peak in the MS1 spectrum. Upon fragmentation, the reporter ions from different variants become distinguishable in the tandem (MS/MS, MS2) mass spectra. Recent developments of Synchronous Precursor Selection (SPS) methods allow us to isolate multiple fragment ions from the MS2 scans and perform accurate quantitative analysis of the reporter ions in the MS3 spectra (7). Intensities of the reporter ions in the MS2 or MS3 spectra are related to the relative abundance of the peptides (8, 9). Both are subject to interferences, and MS2 spectra suffering from higher ratio compression (10). The peptides and proteins are identified by a database search (11, 12), and the intensities are quantified by computational approaches implemented in data processing tools such as Proteome Discoverer, MaxQuant, OpenMS, or SpectroMine.

This multiplexed approach to quantification increases the sample throughput and decreases the experiment time. Importantly, it also reduces the between-run technical variation that is unavoidable during label-free sequential sample processing and data acquisition (13, 14). The quantitative accuracy can be further improved by acquiring technical replicate MS runs from one mixture. However, in many investigations the number of biological samples and conditions exceeds the number of channels. These investigations require multiple MS runs with distinct biological replicates, and possibly also multiple technical MS runs. Investigations with such nontrivial designs may be unbalanced (*i.e.*, they may have an unequal number of replicates from each condition in each mixture).

In addition to the complexities of the designs, larger-scale experiments introduce challenges in the downstream statistical analysis. The stochastic selection of peptide ions for fragmentation implies that the same ions are not consistently observed even between technical replicate MS runs. The problem is exacerbated when the experiment profiles multiple biological mixtures. Therefore, the resulting data have many missing intensities between MS runs (15, 16). Moreover, multiple spectra of the same peptide ion, and multiple peptides of the same protein, may have varying quantitative patterns within and between MS runs, and require normalization (16). The intensities can be further compromised by ion interference, because of co-isolating and co-fragmenting isobaric ions within the isolation window. This causes underestimation of changes in protein abundance (10, 17). In some channels, the intensities can be missing if the analyte is below the limit of detection. Additional technical variation may come from sample preparation (*e.g.* protein extraction, digestion, and isobaric labeling), run-to-run instrumental response fluctuation, etc (18). A combination of complex experimental designs, missing values, biological and technical variation, and interferences complicates protein-level conclusions, and the detection of differentially abundant proteins between conditions. Although strategies for addressing these challenges have been proposed, analysis of these experiments remains challenging.

This manuscript proposes a general statistical approach for relative protein quantification in MS-based experiments with TMT labeling, and is specifically designed for experiments with multiple conditions, multiple biological replicate runs, multiple technical replicate runs, and unbalanced designs. It is based on a flexible family of linear mixed-effects models that handle complex patterns of technical artifacts and missing values. The approach is implemented in *MSstatsTMT*, a freely available open-source R/Bioconductor package compatible with Proteome Discoverer, MaxQuant, OpenMS, and SpectroMine. Below we present the details of the approach, as well as its evaluation on a controlled mixture, simulated data sets, and three biological investigations.

## EXPERIMENTAL PROCEDURES

This section summarizes the data sets in this manuscript. Additional details, including graphical representations of the experimental designs, are in supplemental Section 1.

##### SpikeIn-5mix-MS2 and SpikeIn-5mix-MS3: Controlled Mixtures

The controlled mixtures were used to evaluate *MSstatsTMT* in situations with known ground truth. However, they lack biological variation, and therefore imperfectly represent real-life investigations.

Experimental design: The controlled mixtures aimed to evaluate the ability of *MSstatsTMT* to deal with nontrivial designs with multiple TMT mixtures and multiple technical replicates. 500, 333, 250, and 62.5 fmol peptides from 48 UPS1 proteins were spiked into 50 µg SILAC HeLa peptides in duplicate. This produced a dilution series corresponding to 1, 0.667, 0.5, and 0.125 times of the highest UPS1 peptide amount (500 fmol). In addition, a reference sample was generated by pooling all four diluted UPS1 peptide samples (286.5 fmol) and combined with 50 µg of SILAC HeLa in duplicate. These ten replicates were labeled with TMT 10-plex reagents, mixed and analyzed by LC–MS/MS. The procedure was repeated five times, to generate a total of five such controlled mixtures. Each mixture was profiled in three MS runs, producing 15 MS runs from 5 TMT mixtures in total. The overall experimental design is shown in supplemental Fig. S1.2.

Data acquisition and processing: Raw data for SpikeIn-5mix-MS2 were acquired from the TMT mixtures using MS2-only strategies. Raw data for SpikeIn-5mix-MS3 were acquired using SPS (7). The data were processed with Proteome Discoverer 2.2.0.388 (Thermo Fisher Scientific) and Mascot Server 2.6.1 (Matrix Science, London, UK). Statistical analyses with Proteome Discoverer were done within the software, using proteins marked as “Master” in the protein report. For all the other statistical analyses, reports from Proteome Discoverer 2.2 containing peptide-spectrum matches (PSM) and reporter ion quantifications was exported to R. The PSM reports contained 6767 proteins for the MS2 data set and 5903 proteins for the MS3 data set. Because protein groups containing both spiked-in and background proteins complicated the calculation of the ground truth fold change, all protein groups with multiple proteins were filtered out. 1207 protein groups were removed from the MS2 data set, and 1000 from the MS3 data set. For the same reason, we also removed spiked-in UPS1 proteins sharing sequence with endogenous SILAC-HeLa proteins (20 were removed from the MS2 data set, and 19 from the MS3 data set). The final data set consisted of 5519 proteins (including 20 UPS proteins) in the MS2 data set, and 4812 proteins (including 21 UPS proteins) in the MS3 data set.

Pairwise comparisons: We evaluated the statistical approaches by their ability to detect changes in the abundance of UPS1 proteins between pairs of conditions. Each condition was labeled with the concentration of the UPS1 proteins, *i.e.* 1, 0.667, 0.5, and 0.125. The pairwise comparisons were labeled as the ratios of the concentrations of the UPS1 proteins, *i.e.*, 0.667/0.5, 1/0.667, 1/0.5, 0.667/0.125, 0.5/0.125, and 1/0.125. Therefore, the true fold changes of the UPS1 proteins in these comparisons were 1.33, 1.5, 2, 5.328, 4, and 8.

##### Simulated Datasets Derived from SpikeIn-5mix-MS3

To evaluate *MSstatsTMT* in situations with both biological variation and known ground truth, we created two synthetic data sets by adding biological variation to SpikeIn-5mix-MS3.

SpikeIn-5mix-3TechRep-MS3-Sim: We simulated a data set with the same design as SpikeIn-5mix-MS3. The data set consisted of five mixtures, each profiled with three technical replicate MS runs. The data set was simulated by, first, summarizing all the spectra of a protein in SpikeIn-5mix-MS3 with *MSstatsTMT* as described below, and then adding to the protein summaries in each mixture a simulated random biological variation. Specifically, denote $Y_{mtcb}$ the protein abundance in mixture *m* and technical MS run *t*, in the channel containing biological replicate *b* of condition *c*. The simulated protein abundance $Z_{mtcb}$ was generated as $Z_{mtcb}=Y_{mtcb}+\varepsilon_{mcb}$, where $\varepsilon_{mcb}{∼}^{\mathrm{iid}}N\left(0,\sigma_{S}^{2}\right)$ The same random term was added to all the technical replicates of a subject. We generated datasets with five values $\sigma_{s}=\{0.05,0.1,0.15,0.2,0.4\}$ motivated by the biological investigations in this manuscript.

SpikeIn-15mix-MS3-Sim: We simulated another data set with the same number of runs (15 MS runs total), but now including a larger number of biological replicates and no technical replicates. The simulated random biological variation was added to the protein summaries in SpikeIn-5mix-MS3 as described above.

##### TKO-1mix: A Triple Knockout Proteomics Standard

This investigation had a single MS run and a balanced design.

Experimental design: In the study by Paulo *et al.* (19), three highly abundant proteins (Met6, Pfk2, and Ura2) were knocked out, one at a time, from three yeast proteomes. Each yeast deletion strain was labeled by TMT reagent three times, and the strains were combined in a single TMT 9-plex mixture.

Data acquisition and processing: The mixture was profiled using SPS in a single MS run. The raw data from the original manuscript were re-analyzed for this study using Mascot Server 2.6.2 and Proteome Discoverer 2.2.0.388. All the post-processing steps were the same as in the SpikeIn-5mix data sets. The PSM report contained 1067 proteins. 147 protein groups containing multiple proteins were filtered out, such that the final data set consisted of 919 proteins.

Pairwise comparisons: We evaluated the statistical approaches by their ability to detect changes in the abundance of the knockout proteins between pairs of deletion strains. Each condition was labeled with the knockout protein, *i.e.* ΔMet6, ΔPfk2, and ΔUra2. We performed the pairwise comparisons: ΔMet6-ΔPfk2, ΔMet6-ΔUra2, and ΔPfk2-ΔUra2. The knockout proteins were viewed as true positive changes. Because the knockouts could affect the abundances of the other proteins, the full extent of true positives was unknown.

##### Human-3mix-balanced: Breast Cancer Samples with Fractionation

In this investigation, 27 biological replicates were allocated to 3 TMT mixtures in a balanced design.

Experimental design: Djomehri *et al.* (20) allocated 15 MBC (metaplastic breast carcinoma) samples, 6 TNBC (triple-negative breast cancer) samples, and 6 normal adjacent breast (Control) samples into three TMT 10-plex mixtures. Each mixture had one reference channel, containing a pool of all the 27 samples.

Data acquisition and processing: Each TMT mixture was separated into eight fractions and profiled using SPS, producing a total of 24 MS runs. Raw data were analyzed with MSFragger (v20181128) and Philosopher toolkit (v20181128, github.com/Nesvilab/philosopher) by the authors of the original manuscript. The PSM files were exported to R, for the same post-processing as in the SpikeIn-5mix datasets. If a peptide ion was present in multiple fractions, it was only kept in the fraction where it had the highest mean intensity. If the peptide ion had the same highest mean intensity in multiple fractions, it was only kept in the fraction where it had highest maximal intensity. After merging the nine fractions, the final data set consisted of 5763 proteins. Because the PSM files did not contain protein groups, no additional filtering was necessary.

Pairwise comparisons: We evaluated the statistical approaches by their ability to detect changes in abundance between pairs of conditions across the mixtures. The three conditions were labeled MBC, TNBC, and Control. The pairwise comparisons were MBC-Control, TNBC-Control, and MBC-TNBC. Because the data set is a biological investigation, the true positives were unknown.

##### Mouse-3mix-unbalanced: Mouse Tissue Mixture with Fractionation

In this investigation, 20 biological replicates were allocated to 3 TMT mixtures in an unbalanced design.

Experimental design: Plubell *et al.* (21) subjected twenty mice to either low-fat or high-fat diets for either a short (8 weeks) or long (18 weeks) duration. Five mice were subjected to each combination of low (LF) or high fat (HF) and short or long-term diet. Samples from epididymal adipose tissue of the mice were randomly allocated to three TMT 10-plex mixtures. Each mixture included two reference channels with pooled samples, containing a proportion of peptides from each sample. Some channels were unused and resulted in an unbalanced design.

Data acquisition and processing: Each TMT mixture was separated into eight fractions and profiled using SPS, producing a total of 27 MS runs. Raw data were downloaded from ProteomeXchange (identifier PXD005953) and re-analyzed for this study with Mascot Server 2.6.2 and Proteome Discoverer 2.2.0.388. All the post-processing steps were the same as in the Human-3mix-balanced data set. The PSM report from Proteome Discoverer contained 5823 proteins. 730 protein groups containing multiple proteins were filtered out, such that the final data set consisted of 4713 proteins.

Pairwise comparisons: We evaluated the ability of the statistical approaches to detect changes in abundance between pairs of conditions across the mixtures. The four conditions were labeled Long_HF, Long_LF, Short_HF, and Short_LF. The pairwise comparisons were Long_HF-Long_LF, Short_HF-Short_LF, Long_HF-Short_HF, and Long_LF-Short_LF. Because the data set is a biological investigation, the true positives were unknown.

## RESULTS

##### Existing Statistical Methods for Experiments with Isobaric Labeling

This section reviews the existing statistical analysis strategies for experiments with isobaric labeling, and partitions them into a series of common steps summarized in the rows of Table I. Specifically, *spectrum-level normalization* reduces artifacts of sample preparation or mass analysis at the level of reporter ion intensities. *Protein summarization* takes as input all reporter ion intensities (or their ratios) of a protein in a run and aggregates them into a single estimate of protein abundance per channel per run. *Protein-level normalization* reduces the technological artifacts in the protein summaries. *Statistical modeling and inference* quantifies the sources of systematic and random variation for each protein, and tests proteins for differential abundance. Various workflows approach these steps in various ways. The steps can be applied locally (*i.e.*, separately within a spectrum or a protein), or globally (*i.e.*, simultaneously to all spectra or proteins in a run), and may or may not rely on a reference channel with constant protein abundance across the runs. Not every workflow uses every step.

**Table I:** Representative workflows for differential analysis of mass spectrometry experiments with isobaric labeling. All the four methods take as input the same PSM report from a data processing tool. Rows in the Table classify the statistical analyses into a series of common steps. Columns in the Table are representative workflows, adapted from the corresponding publications or open-source code. Local steps are applied within a spectrum or a protein. Global steps are applied to all spectra or proteins

Method	Ratio+Median+Limma Adapted from Herbrich *et al.* [22] and Kammers *et al.* [23]	Sum+IRS+edgeR Adapted from Plubell *et al.* [21]	Proteome Discoverer 2.2 User Guide Book [51] Adapted from McAlister *et al.* [7]	MSstatsTMT This Manuscript
Spectrum-level normalization	Local ratio-based normalization: *log*_2_ transform the intensities; for each spectrum, calculate *log*_2_ ratio	None	None	Global median normalization: *log*_2_ transform the intensities; equalize the median of the *log*_2_ intensities across all spectra, channels and MS runs
	- without reference channel: subtract the median of *log*_2_ intensities of all channels in the spectrum			
	- with reference channel: subtract *log*_2_ intensity of the reference channel			
Protein summarization	Median summarization: for each protein and each channel, estimate protein ratio as the median of all the *log*_2_ ratios of all the spectra of the protein	Sum summarization: for each protein and each channel, estimate protein summary as the sum of all the spectrum intensities on the original (not log) scale	Sum summarization: for each protein and each channel, estimate protein summary as the sum of all the spectrum intensities on the original (not log) scale	Tukey's median polish: for each protein and each run, impute missing values with Accelerated Failure Time model and estimate protein summary in each channel with Tukey's median polish
Protein-level normalization	Global zero median normalization: for each run and each channel, subtract the median of all the *log*_2_ ratios across proteins, such that the median ratio of each channel is zero	- Remove proteins with missing summaries	For each run:- Global equal sum normalization: sum the summaries of all proteins in each channel, and equa lize the sums over all channels and runs- Local protein scaling: scale the normalized summaries of each protein to have an average of 100.	Local normalization with reference channel: for each protein, equalize the *log*_2_ protein summaries in the reference channel of each MS run to their median across all the runs
		- Global equal sum normalization: sum the summaries of all proteins in each channel, and equalize the sums over all channels and runs- Local IRS normalization with reference channel: scale the normalized summaries in the reference channel in each run to equalize their geometric means across runs		
Statistical modeling and inference	linear model with limma. The linear model includes fixed run effect and condition effect.	Negative Binomial regression edgeR with library size correction; uses subsets of data with pairs of conditions	one-way ANOVA	linear mixed-effects model fit simpler model for proteins where parameters of full model are not estimable
Applicable experimental designs	- single mixture with single technical replicate MS run	- treat every design as single mixture with single technical replicate MS run	- treat every design as single mixture with single technical replicate MS run	- single mixture with single technical replicate MS run
	- multiple mixtures with single technical replicate MS run OR single mixture with multiple technical replicate MS runs			- single mixture with multiple technical replicate MS runs
				- multiple mixtures with single technical replicate MS run
				- multiple mixtures with multiple technical replicate MS runs
Implementation	adapted from code in Kammers *et al.* [52] to handle multi-group designs	adapted from code in Wilmarth *et al.* [53]	proprietary, Proteome Discoverer 2.2	R/Bioconductor package *MSstatsTMT*

Columns in Table I summarize two representative workflows that we call *Ratio*+*Median*+*Limma* (22, 23) and *Sum*+*IRS*+*edgeR* (21). We selected these workflows because they represent two main commonly used approaches (ratio-based and sum-based), have an open-source implementation, and are compatible with multiple data processing tools. Additionally, the table summarizes the statistical analysis workflow of *Proteome Discoverer* 2.2, based on its user guidebook and the method by McAlister *et al.* (7). We overview the steps of these workflows below and illustrate each step in supplemental Section 2. Whenever possible, we expand the discussion to other approaches that focus on each step.

Spectrum-level normalization: We loosely classify spectrum-level normalizations into two groups. The first group uses *local ratio-based normalization*, as exemplified by *Ratio*+*Median*+*Limma*. If a reference channel is available, for each spectrum the method subtracts from the *log_2_* intensities of the endogenous channels the *log_2_* intensity of the reference channel. In absence of a reference channel, the method assumes a constant protein abundance across the mixtures and replaces the reference channel with the median of *log_2_* intensity in the spectrum (23). The resulting *log_2_* ratios are centered around 0 (and the ratios on the original scale are centered around 1), as illustrated in supplemental Fig. S2.1A.

The second group of methods use *global spectrum-level normalization*. These methods do not calculate ratios but assume constant total protein abundance across all the spectra, channels, and runs. For example, variance stabilizing normalization (VSN) transforms the reporter ion intensities to roughly equalize their variance over the entire intensity range. Originally designed for transcriptomics, the method was specifically adapted to proteomic experiments with isobaric labeling (24, 25, 26). *CONSTANd* (27) was explicitly designed for multiplexed proteomic experiments as an instance of constrained optimization. For each run, the method constructs a data matrix where rows are spectra, columns are channels, and entries are intensities of the reporter ions in that run. The method estimates a normalized version of the data matrix with maximal similarity to the original matrix while satisfying two equality constraints. The first constraint ensures that the summation of each row of the normalized matrix is equal to 1. The second constraint ensures the summation of each column is equal to a value determined by the number of spectra and channels in a run.

Protein summarization: Statistical methods for protein summarization can also be loosely classified into two groups. The first group assumes that all the spectra represent the protein abundance equally well. *Ratio*+*Median*+*Limma* employs Median summarization, which for each protein and each channel estimates protein-level *log_2_* as the median of the *log_2_* ratios of the spectra (22, 23). In contrast, Sum+IRS+edgeR (21) and McAlister *et al.* (7) (adapted by *Proteome Discoverer* 2.2) use *Sum summarization*, which sums the reporter ion intensities of all the spectra on the original (*i.e.* not log-transformed) scale. The methods are illustrated in supplemental Fig. S2.2. Other methods include Tukey's median polish, which considers all the channels in an MS run simultaneously, takes as input the *log_2_* reporter ion intensities of the protein across all the channels, and iteratively fits a two-way robust additive model (28). The second group of protein summarization methods assigns different weights to different spectra of the protein and estimates protein abundance with a weighted average of the reporter ion intensities or ratios of its spectra. Summarization methods in this group differ in how they estimate the weights. For example, the R package *isobar* (29) calculates ratios of intensities between pairs of channels in a spectrum, estimates the noise variance of the ratios, and uses the inverse of the variances as the ratios' weights. The output of the procedure is not a summary of protein abundance per channel, but a ratio of protein abundances between pairs of channels or pairs of conditions. Method *iPQF* (30) in the R package MSnbase (28) estimates the weights of the spectra based on multiple spectral characteristics, such as peptide mass and charge.

Protein-level normalization: Many recent methods apply normalization to protein summaries, and many require at least one reference channel in each MS run. *Sum*+*IRS*+*edgeR* employs two normalization procedures as illustrated in supplemental Fig. S2.3. The first is a *global equal sum normalization*, which sums the summaries of all proteins in each channel on the original scale, and equalizes the sums over all channels and runs. The second is a *local Internal Reference Scaling (IRS)* normalization (21), which normalizes each protein separately. For each protein, IRS normalization first calculates a geometric mean of the normalized protein summaries in the reference channel across the runs. Next, the method calculates a scale factor, *i.e.* a ratio of the protein summary in the reference channel of each run to the geometric mean above. Finally, the protein summary in every channel is multiplied by the scale factor of its run. An alternative approach in (22) calculates the ratio of a protein summary in a channel to the protein summary in the reference channel of the run.

The second group of methods does not use a reference channel at this stage. For example, *Ratio*+*Median*+*Limma* implements a *global zero median normalization* (22, 23). For each run and channel, it subtracts the median of all the protein-level *log_2_* ratios, to set to zero the median over all the protein summaries in the channel. McAlister *et al.* (7) use a *global equal sum normalization* that equalizes the sum of the protein summaries across the channels and runs. *Proteome Discoverer* 2.2 supplements the global equal sum normalization with *protein scaling*, which scales the normalized summaries across each protein to generate the protein ratios with a total or average of 100. The methods are illustrated in supplemental Fig. S2.3.

Statistical modeling and testing for differential abundance: Most statistical methods for detecting differentially abundant proteins are applied after protein summarization and normalization. The simplest approach, implemented in Proteome Discoverer 2.2, fits a *one-way Analysis of Variance (ANOVA)* to all the protein summaries from all the runs (7). Alternatives use statistical methods originally designed for transcriptomics, such as R/Bioconductor packages *limma* (31) and *edgeR* (32).

More complex statistical modeling is required for experiments with multiple MS runs and missing values. *Ratio*+*Median*+*Limma* (23) extends *limma* to explicitly account for multiple MS runs. The method takes as input the *log_2_* protein ratios produced by normalization and summarization, and fits a two-way additive linear model with a fixed group effect and a fixed MS run effect, which does not distinguish between biological and technical replicate MS runs. It then uses the Empirical Bayes procedure in *limma* to combine the estimates of random variation across all the proteins in a moderated t-statistic (33). Although the original implementation of *limma* did not allow proteins with missing values, more recent *limma* 3.44 includes proteins with missing values into analyses. D'Angelo *et al.* (34) expanded the use of *limma* by imputing missing values within an MS run, and excluding peptide ions that were completely missing in at least one MS run. In experiments with multiple MS runs, this exclusion significantly reduced the number of proteins that can be tested for differential abundance. *Sum*+*IRS*+*edgeR* (21) uses edgeR, originally designed for transcriptomic experiments. The model is primarily appropriate for experiments that generate data in form of discrete counts because it assumes a negative binomial distribution. The implementation is limited to two conditions (or subsets of the data set with pairs of conditions), requires an additional normalization with respect to the total protein abundances in a sample (called library size), and removes proteins with any missing values.

Several statistical methods take as input reporter ion intensities before protein summarization. Paulo *et al.* (35) first summarize the spectra at the peptide level, and use the summaries as input to an additive linear model that includes a group effect and a peptide effect, but ignores a run effect. Oberg *et al.* (15) take as input MS/MS spectra, and fit a linear mixed-effects model that decomposes the variation in the reporter ion intensities into contributions from multiple sources, including multiple MS runs. The model has many parameters, is limited to balanced designs, and requires computationally intensive procedures such as stage-wise or iterative regression (36).

### Proposed Statistical Methodology

##### Input to MSstatsTMT and Notation

**Fig. 1:**
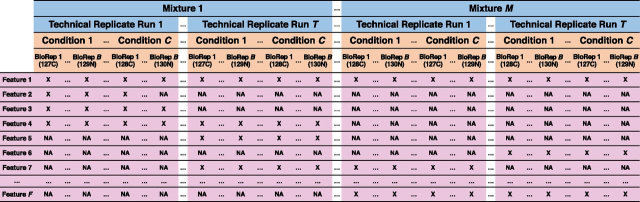
**Representative design of a proteomic experiment with isobaric labeling, for one protein.** The experiment has *M* mixtures, *T* technical replicates MS Runs per biological mixture, *C* conditions and *S* biological replicates per condition and mixture, resulting in *MTCB* observations per feature. The protein has *F* features. Subjects in a mixture are randomly quantified with isobaric channels (e.g., 127C and 129N). In the language of experimental design, an MS run is a whole plot (in blue), each combination of conditions and biological replicates is a subplot (in orange), and a feature is a sub-subplot (in purple). The symbol X in each cell denotes the *log*_2_ reporter ion intensity of the observed feature and NA denotes missing feature intensity. When a feature is not identified in one MS run, the values of all the corresponding cells are NA. For example, Feature 3 is only identified in Technical Replicate Run 1 of Mixture 1.

Fig. 1 outlines a representative design of a proteomic experiment with isobaric labeling, and the input to *MSstatsTMT* for one protein. The experiment has *m* = 1,…, *M* biological *Mixtures*. Each mixture contains samples from distinct biological subjects, labeled with isobaric tags (*e.g.* TMT 10- or 11-plex). Each mixture is profiled in *t* = 1,…, *T Technical replicate* MS runs. Therefore, the experiment has a total of M × T MS *Runs*. In practice, *MSstatsTMT* can be applied to any number and type of technical replicates. For example, technical replicates can be separately digested and randomly labeled to reflect the variation because of digestion and labeling. Biological replicates from different conditions can be assigned to different channels in each MS run.

This manuscript focuses on a group comparison design,*i.e.* a design with *c* = 1,…, *C Conditions* (such as treatments, or disease types), where each condition is represented by different subjects. Each MS run consists of *b* = 1,…, *B Biological replicates (BioRep)* from each of the *C* conditions. Thus, each MS run has B × C distinct biological replicates. For simplicity, below we refer to each column in Fig. 1 as a *Channel*. In the example of Fig. 1, the experiment has *MTCB* channels.

In each MS run, the protein is represented by *f* = 1,…, *F Features*. The features are MS2 or MS3 spectra identified by a search engine such as Mascot or Sequest. In each run and each channel, each feature is quantified by a *log_2_*-transformed intensity of the reporter ion (defined as the height of the reporter ion peak, or any other measurement) by a data processing tool such as Proteome Discover, MaxQuant, or SpectroMine and denoted *X_mtcbf_*. The *log_2_* transformation is important, because measurements on the log scale conform more closely to the Normal distribution (37) and better satisfy the statistical modeling assumptions.

Fig. 1 represents a balanced design, *i.e.*, a design where all the conditions in a mixture have the same number of biological replicates. In practice, the experiment design can be unbalanced and can contain a different number of biological replicates within a mixture and a condition, and a different number of technical replicates per mixture. *MSstatsTMT* applies to these situations.

The data structure in Fig. 1 can also be unbalanced because of missing feature intensities. Occasionally, a reporter ion channel can be missing within a feature. More frequently, missing features arise when peptide ions are inconsistently identified between the MS runs and especially between the mixtures. Some of the observed intensities can be compromised by interferences and thus become outliers.

##### Statistical Motivation behind MSstatsTMT

Optimal statistical analysis is always motivated by the experimental design (38). Experiments with isobaric labeling impose two restrictions on data collection. First, all the conditions and subjects in a mixture are quantified simultaneously within an MS run, and are therefore simultaneously affected by the random artifacts in that run. Second, all the features of a protein are quantified simultaneously within a run. In the statistical language, such two-layer restrictions are known as split-split-plot designs (39, 40), where a MS run is a whole plot, each combination of conditions and subjects is a subplot, and a feature is a sub-subplot. In experiments with isobaric labeling, comparison of interest, *e.g.* conditions, are at the level of the subplot.

In the general statistical literature, the split-split-plot design is optimally represented by a linear mixed-effects model with multiple variance components, described in supplemental Section 3.1 (39, 40). Unfortunately, the estimation of these models is not scalable to large-scale datasets with many proteins and runs and is difficult to extend to cases with missing values and outliers. At the same time, in a special case of balanced design, parameter estimation and model-based inference in these models only depend on the summary of the reporter ion intensities per channel and run. In other words, in balanced designs it is sufficient to first summarize the *log_2_*-intensities of the reporter ions over all the features of a protein in a run (*i.e.* in the sub-subplot), and then fit a simplified protein-level model that only involves the whole plot and the sub-plot.

Based on this insight from balanced designs, we propose a workflow, called *MSstatsTMT*, that generalizes the inference to complex designs, many proteins and multiple data processing tools. *MSstatsTMT* separates spectrum-level normalization, protein summarization and protein-level normalization, and then uses normalized protein summaries as input to statistical modeling and inference. In the special case of balanced designs, conclusions from the proposed approach are equivalent to the conclusions from the optimal general model in supplemental Section 3.1.

##### Protein Summarization and Normalization in MSstatsTMT

**Fig. 2:**
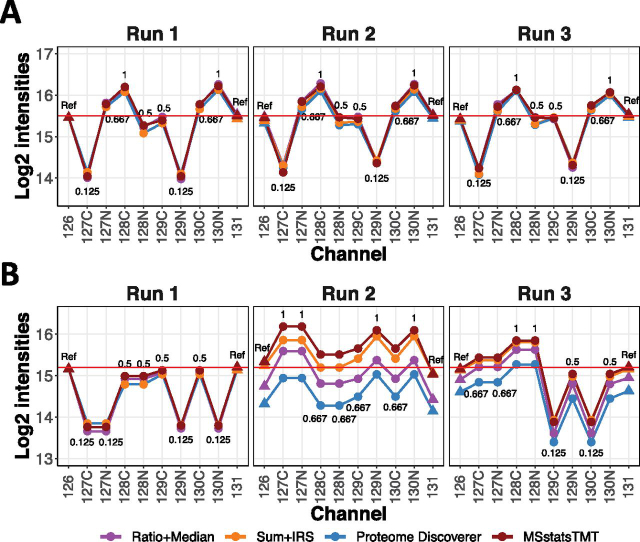
**Spectrum-level normalization, protein summarization and protein-level normalization in representative workflows in**Table I, **in a hypothetical experiment with two reference channels and three runs.** The individual steps are detailed in supplementary Section 2. Each panel is a MS run. *x*-axis: TMT channels. *y*-axis: *log*_2_ intensity. Colored dots are *log*_2_ protein intensities summarized and normalized by each workflow, labeled with the true abundance in the respective channel and mixture. Triangle dots indicate reference channels. To make the scale of the protein summaries comparable between workflows, the normalized protein intensities reported by *Sum*+*IRS*+*edgeR* and *Proteome Discoverer* were *log*_2_ transformed, and equalized to the protein summaries in Channel 126 of Run 1. Red horizontal line indicates the median of the protein summaries in the reference channels across the runs, as estimated by *MSstatsTMT*. *A*, Balanced design, where each run has an equal number of replicates from each condition. All the workflows equalized the reference channels between the runs, and reported similar normalized protein summaries. *B*, Unbalanced design, where each run has a different number of replicates from each condition. Normalizations by *Ratio*+*Median*+*Limma* and *Proteome Discoverer* failed to eliminate undue variation between reference channels and compressed the differences in protein summaries across conditions and runs. Normalizations with respect to the reference channels by *Sum*+*IRS*+*edgeR* and *MSstatsTMT* avoided this artifact.

Each step of *MSstatsTMT* is summarized in Table I, and illustrated in supplemental Fig. S2.1. The combined outcome of protein summarization and normalization is illustrated for one example protein in Fig. 2.

Global median normalization between channels: This step simultaneously considers all the features identified in the experiment. Similarly to *isobar* (29) and to normalizations used in label-free quantification (41), *MSstatsTMT* assumes that the total abundance of the analytes is equal across all the channels and runs. Therefore, *MSstatsTMT* applies a global equal median normalization between channels to account for differences in labeling efficiency and other technical artifacts. As illustrated in supplemental Fig. S2.1D, *MSstatsTMT* equalizes the median of the reporter ion intensities across all the channels and MS runs.

Protein summarization: This step and all the subsequent steps of *MSstatsTMT* consider one protein and one MS run at a time. It focuses on the sub-subplot aspect of the experimental design (supplemental Section 3.1), and summarizes the *log_2_* intensities of the features in each channel and MS run while accounting for missing and outlying feature intensities. This summarization is identical to the summarization used for label-free experiments in MSstats (42). Specifically, it fits the observed intensities of a protein to a two-way model.

(1) $$X_{mtcbf}=\mu_{mt}+Feature_{f(mt)}+Channel_{b(mtc)}+\varepsilon_{mtcbf}$$

$$\Sigma_{f}Feature_{f(mt)}=0,\Sigma_{cb}Channel_{b(mtc)}=0$$

Assuming that the missing feature intensities primarily arise from low-abundant analytes, *MSstatsTMT* extends the model above with the Accelerated Time Failure assumption (43) and imputes the missing feature intensities within each MS run. To impute an intensity of a feature MSstatsTMT requires at least one nonmissing channel for the same feature in that run, and at least one nonmissing feature from the same protein in the same channel in that run. If the entire feature was not quantified in a run, it is left missing. If the entire protein was not quantified in a channel, all the intensities from that protein in that channel are left missing. Next, to eliminate the undue influence of outliers, *MSstatsTMT* re-estimates the parameters of the additive model from the observed and the imputed values with the Tukey's median polish (44). Finally, *MSstatsTMT* summarizes the protein abundance *Y_mtcb_* in a channel and in a run containing biological replicate *b* of condition *c* profiled by technical MS run *t* of mixture *m* as

(2) $$Y_{mtcb}={\hat{\mu }}_{mt}{+\hat{Channel}}_{b(mtc)}$$

The values *Y_mtcb_* are the sub-subplot level summaries in this design.

Local protein-level normalization with reference channel: This second normalization takes the protein summaries in Eq. 2 as input. Because different features of a protein are typically identified in different MS runs, and because the features differ in ionization efficiency and other biochemical properties, the protein summaries are not comparable between runs. To account for this, *MSstatsTMT* relies on the presence of at least one reference channel. The reference channel lacks biological variation and reflects technological artifacts (such as different labeling and ionization efficiency). For each protein, *MSstatsTMT* equalizes the protein summaries in the reference channel of each MS run to the median of the reference channels between the runs. It then applies the corresponding shifts to the protein-level summaries in the remaining channels of each run, as illustrated in supplemental Fig. S2.3D. If the design includes multiple reference channels per MS run, *MSstatsTMT* starts by averaging the protein summaries of the reference channels.

The local protein normalization by *MSstatsTMT* is similar in spirit to that of IRS normalization, but is different in that it is applied to the og-scaled protein summaries. It is equivalent to calculating log-ratios between the endogenous and the reference channels and rescaling the log-ratios to a common median value. The local protein normalization by *MSstatsTMT* is like the approach by Kammers *et al.* (23) in *Ratio*+*Median*+*Limma* in balanced designs. However, the results of *MSstatsTMT* and *Ratio*+*Median*+*Limma* differ substantially when MS runs contain different number of replicates from each condition. Fig. 2 illustrates that in unbalanced designs, normalization without a reference channel can remove the true biological signal. Normalization with respect to a reference channel avoids this artifact.

##### Statistical Modeling and Inference in MSstatsTMT

The normalized protein-level summaries are used as input to statistical modeling. For experimental designs with multiple biological replicates, multiple mixtures, and multiple technical replicates (such as in Fig. 1), *MSstatsTMT* fits the following model:

(3) $$Y_{\mathrm{mtcb}}=\mu +{\mathrm{Mixture}}_{m}+\mathrm{TechRep}{\left(\mathrm{Mixture}\right)}_{t\left(m\right)}+{\mathrm{Condition}}_{c}+{\mathrm{Subject}}_{\mathrm{mcb}}+\varepsilon_{\mathrm{mtcb}}$$

Where

$${\mathrm{Mixture}}_{m}{∼}^{\mathrm{iid}}N(0,\sigma_{M}^{2}),\mathrm{TechRep}{\left(\mathrm{Mixture}\right)}_{t(m)}{∼}^{\mathrm{iid}}N(0,\sigma_{T}^{2}),\Sigma_{c=1}^{C}{\mathrm{Condition}}_{c}=0,$$

$${\mathrm{Subject}}_{\mathrm{mcb}}{∼}^{\mathrm{iid}}N\left(\text{0,}\sigma_{s}^{\text{2}}\right),\varepsilon_{\mathrm{mtcb}}{∼}^{\mathrm{iid}}N\left(\text{0,}\sigma^{\text{2}}\right)$$

The term *Subject* represents biological replicates, with the convention that each biological replicate has a unique identifier across mixtures and conditions. *Mixture* and *TechRep*(*Mixture*) distinguish technical variation between mixtures, and between replicate MS runs of a same mixture. ε represents the technical variation that is not explained by *Mixture* and *TechRep*(*Mixture*). Supplemental Section 3 provides the methodological justification, and supplemental Section 4 the empirical justification behind this model. As the result of detailed modeling of systematic sources of variation, random errors at the level of protein summaries $\varepsilon_{mtcb}$ can be assumed independent and nonsystematic. When the experimental design does not include replicates for all the sources of variation in Fig. 1 some terms in Eq. 3 are not estimable. In this case *MSstatsTMT* fits simpler models as described in supplemental Section 3.

Parameters of the model are estimated using restricted maximum likelihood. Because the number of biological replicates in each condition is often small, *MSstatsTMT* adopts Empirical Bayes moderation of the standard errors, as proposed in the R package *limma* for analysis of gene expression microarrays (45). Model-based tests for differentially abundant proteins between pairs of conditions is carried by comparing the terms *Condition* (see supplemental Section 3.7 for technical details). Finally, *MSstatsTMT* adjusts the p-values of the tests to account for multiple comparisons between the proteins by the method of Benjamini-Hochberg FDR (46).

##### Implementation of MSstatsTMT

We implemented this workflow for general group comparison designs in the open-source R/Bioconductor package *MSstatsTMT* (47). *MSstatsTMT* includes converters from Proteome Discoverer, MaxQuant, OpenMS, and SpectroMine. In addition to formatting the data, the converters construct spectral features as follows. The converters remove spectra with an excessive number of missing reporter ion intensities, or peptide identifications shared by multiple proteins. If multiple spectra have the same peptide ion identification, the converters only retain a single “best” spectrum with the minimal number of missing values, highest intensity, or lowest interference score. If the experiment contains fractions, and a peptide ion is present in multiple fractions, the peptide ion is only kept in the fraction where it has the highest mean intensity. If the peptide ion has the same highest mean intensity in multiple fractions, it is only kept in the fraction where it has highest maximal intensity. Proteins with more than one summary value in more than one condition are retained for the downstream statistical analysis.

The missing value imputation and protein summarization steps in *MSstatsTMT* rely on functionalities in the R package MSstats (42). Statistical modeling, inference and hypothesis testing relies on the functionalities in the R packages *lme4* (48) and *lmerTest* (49). The Empirical Bayes moderation relies on the functionalities in the R package *limma* (31). Analyses of all the datasets in this manuscript were completed in under one hour on a MacBook Pro with Intel Core i5 and 8 GB memory.

### Evaluation

##### Evaluation Strategy

We evaluated the performance of *MSstatsTMT* v1.6.2 while comparing all pairs of conditions in the datasets under “Experimental Procedures”, using workflows in Table I. All workflows except Proteome Discoverer took as input features produced by the *MSstatsTMT* converter. For *Ratio*+*Median*+*Limma*, we selected spectrum-level normalization without using reference channel as recommended by Kammers *et al.* (23). For Proteome Discoverer 2.2, the input and the results of statistical analysis were as reported by the software.

We defined a *testable protein* a protein with enough data to perform a test for differential abundance with a workflow and used the number of testable proteins in each workflow as a criterion for evaluation. Evaluations on the controlled mixtures and on the simulation experiments considered the number of true positives (TP), false positives (FP), true negatives (TN) and false negatives (FN), where the total TP+FP+TN+FT equals to the number of testable proteins specific to each comparison and each workflow. We also considered the empirical false discovery rate (eFDR = FP/(TP+FP)), the sensitivity (TP/(TP+FN)) and the specificity TN/(TN+FP) of detecting differentially abundant proteins among the testable proteins at the FDR = 0.05 cutoff. We further considered area under the ROC curve (AUC), which represents sensitivity *versus* 1-specificity at various FDR-adjusted p-value cutoffs, calculated using R package *pROC* (50). Finally, we compared the estimated fold changes to the true fold changes. Evaluations on biological investigations focused on the number of differentially abundant proteins at FDR = 0.05.

##### Evaluation on Controlled Mixtures

We first evaluated *MSstatsTMT* on controlled mixtures that contained ground truth but lacked biological variation. Because the controlled mixtures had a balanced design, all normalization and summarization methods produced relatively similar results (similarly to the illustration in Fig. 2), and differences in performance were primarily because of statistical modeling and inference.

**Fig. 3:**
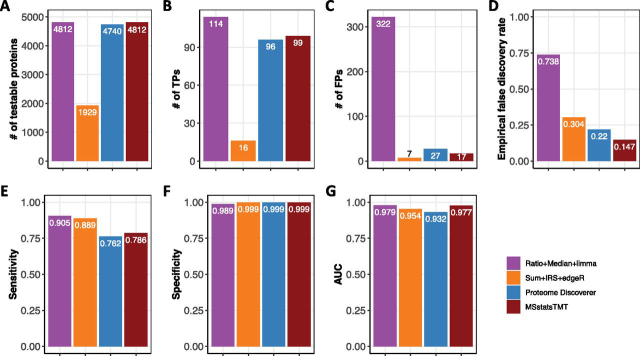
**Detection of differentially abundant proteins in all pairs of conditions in SpikeIn-5mix-MS3 (FDR cutoff of 0.05).** Colors represent statistical modeling and inference methods in Table I. *A*, Number of testable proteins. *B*, Number of true positive differentially abundant proteins. *C*, Number of false positive differentially abundant proteins. *D*, Empirical false discovery rate. *E*, Sensitivity of correctly detecting the spiked-in proteins. *F*, Specificity of correctly detecting the background proteins. *G*, Area under ROC curve (AUC).

MSstatsTMT best balanced the number of true and false positive differentially abundant proteins: Fig. 3 summarizes the performance of the representative workflows in Table I on SpikeIn-5mix-MS3. For investigations without biological variation, *MSstatsTMT* fit the simple model in supplemental Section 3.6. This model is similar (but not identical) to the models fit by *limma* and one-way ANOVA (implemented in *Proteome Discoverer*), and these workflows had a similar number of testable proteins. *EdgeR* fit a different model, which assumed that reporter ion intensities were count data following a Negative Binomial distribution. The inappropriate assumption, combined with subsetting the data set for each pair of conditions, negatively affected the number of testable proteins.

*limma* produced the largest number of both true and false positive differentially abundant proteins, and the largest eFDR. This was because of a combination of the treatment of missing values and of the Empirical Bayes step, which under-estimated the biological variation. Because the data set had a relatively small number of true differentially abundant proteins (21) as compared with the background proteins (4791), all the workflows produced similar sensitivity, specificity and AUC (calculated with respect to their individual number of testable proteins). The performance of one-way ANOVA was close (but slightly worse) than *MSstatsTMT*. Overall, *MSstatsTMT* best balanced the number of true positives and the eFDR.

MSstatsTMT had qualitatively similar performance in MS2 and MS3 data acquisition: supplemental Fig. S5.1 shows that SpikeIn-5mix-MS2 produced more testable proteins than SpikeIn-5mix-MS3, for all the workflows. This led to a larger number of both true and false positives. *MSstatsTMT* reported more true positives as compared with the other workflows while minimizing eFDR, a result consistent with SpikeIn-5mix-MS3. supplemental Fig. S5.2 shows that all the workflows underestimated the true fold changes of the spiked proteins to some extent in both MS2 and MS3 acquisitions. This may be because of the low abundance (<1%) of spiked-in peptides as compared with the back- ground peptides (approximately 14 ng as compared with 2 μg for the UPS1 and SILAC-HeLa peptides, respectively). Despite the larger number of proteins in MS2, its ratio compression was more severe, confirming that MS3-based quantification can help alleviate ratio compression.

##### Evaluation on Controlled Mixtures with Simulated Source of Biological Variation

To evaluate *MSstatsTMT* in experiments in presence of biological variation, we simulated various amounts of biological variation added to SpikeIn-5mix-MS3 as described in EXPERIMENTAL PROCEDURES. As before, because of the balanced nature of the designs, differences in performance primarily come from statistical modeling and inference.

**Fig. 4:**
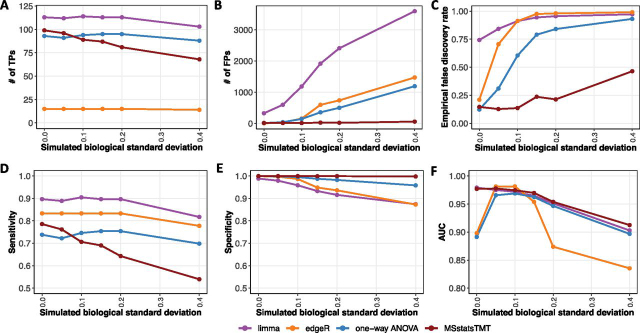
**Detection of differentially abundant proteins in all pairs of conditions in SpikeIn-5mix-3TechRep-MS3-Sim (FDR cutoff of 0.05).** Colors represent statistical modeling and inference methods in Table I. X-axis: simulated biological standard deviation (standard deviation = 0 corresponds to the original controlled mixtures SpikeIn-5mix-MS3). *A*, True positive differentially abundant proteins. *B*, False positive differentially abundant proteins. *C*, Empirical false discovery rate. *D*, Sensitivity of detecting the spiked-in proteins. *E*, Specificity of detecting the background proteins. *F*, Area under ROC curve (AUC).

MSstatsTMT accurately characterized biological variation in investigations with both biological and technical replicates: The simulated data set SpikeIn-5mix-3TechRep-MS3-Sim had five biological mixtures, three technical replicate MS runs per mixture, and a balanced design. Fig. 4 summarizes the performance of the workflows. *MSstatsTMT* fit the model in Eq. 3, which distinguished these sources of variation. In contrast, the models in *limma*, *edgeR* and one-way ANOVA (implemented in *Proteome Discoverer*) did not have enough flexibility to distinguish biological and technical variation. They combined the variation from these two sources, which lead to over-estimation of the degrees of freedom and under-estimation of the standard error and increased the false positive differentially abundant proteins.

**Fig. 5:**
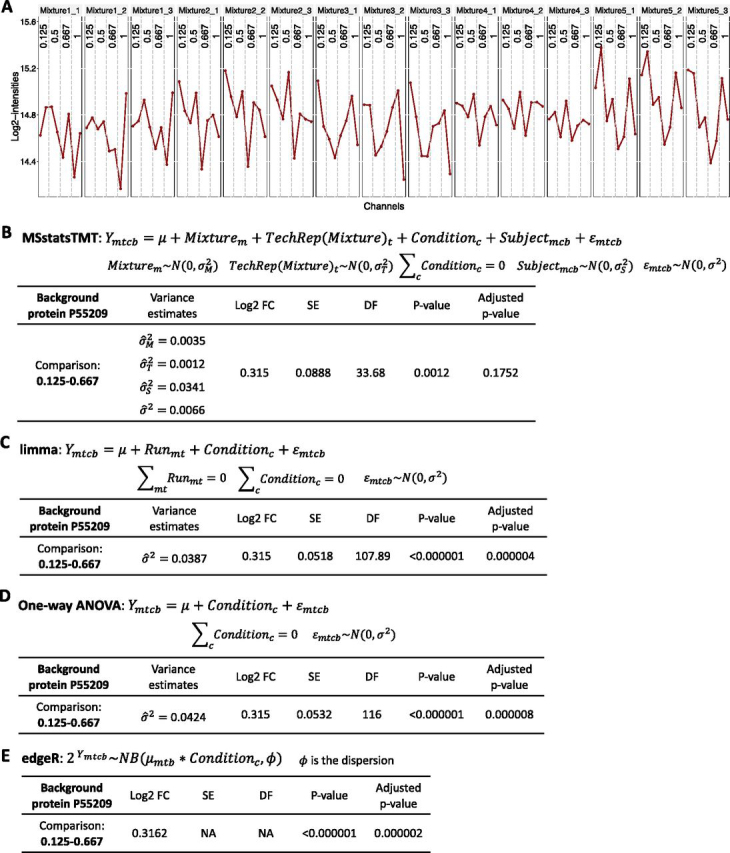
**Background protein P55209 (Nucleosome assembly protein 1-like 1, NP1L1 human) from SpikeIn-5mix-3TechRep-MS3-Sim.** The experiment had a total of 15 runs, with 5 biological mixtures and 3 technical replicates each. The simulated biological standard deviation was set to 0.2, and therefore the true variance of the term *Subject* was 0.04. The protein did not have any missing protein summary values, and therefore was testable with all the workflows. *A*, Normalized *log*_2_ protein-level summaries by *MSstatsTMT*. *x*-axis: TMT channels. *y*-axis: *log*_2_ intensity. Each panel represents one MS run. Labels inside each panel are conditions of the corresponding channel and mixture. *B*, *MSstatsTMT*: model and model-based inference comparing condition 0.125 and 0.667. Because this is a background protein, the true *log*_2_ fold change is 0. *MSstatsTMT* separated the sources of biological and technical variation. The estimate of biological variation was 0.0341 (close to the true value of 0.04). The protein was not found differentially abundant. *C*, *limma*: model and model-based inference comparing condition 0.125 and 0.667. *limma* did not separate the biological and the technical variation. It reduced the standard error and inflated the degrees of freedom associated with the comparison and resulted in a false positive conclusion. *D*, *One-way ANOVA* implemented in *Proteome Discoverer*: same conclusion as in (*C*). *E*, *EdgeR*: model and model-based inference comparing condition 0.125 and 0.667 *EdgeR* was unable to estimate the standard error of the estimated fold change, but reported the protein as differentially abundant.

Fig. 5 further details the differences between the workflows in the case of one example protein. In this example, *MSstatsTMT* was the only method that separated the sources of biological and technical variation, accurately estimated the standard error and the degrees of freedom and did not result in a false positive detection of differential abundance. Overall, in presence of both biological and technical replicates, *MSstatsTMT* best balanced the number of true positives and the eFDR.

MSstatsTMT best balanced true and false positive differentially abundant proteins in investigations with many biological replicates: We further simulated an experiment SpikeIn-15mix-MS3-Sim with the same number of runs, but no technical replicates. Instead, it contained up to 15 distinct biological mixtures, corresponding to up to 30 biological replicates per condition.

**Fig. 6:**
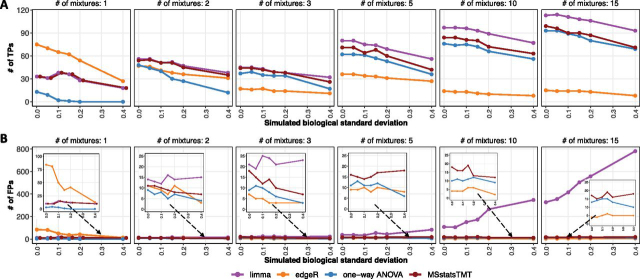
**Detection of differentially abundant proteins in all pairs of conditions in SpikeIn-15mix-MS3-Sim (FDR cutoff of 0.05).** SpikeIn-15mix-MS3-Sim experiment simulated 15 biological mixtures and no technical replicates. Panels in the figure represent randomly selected subsets of 1, 2, 3, 5, 10 mixtures, and 15 mixtures. Colors represent statistical modeling and inference methods in Table I. *x*-axis: simulated biological standard deviation (standard deviation = 0 corresponds to the original controlled mixtures SpikeIn-5mix-MS3). *A*, Number of true positive differentially abundant proteins. *B*, Number of false positive differentially abundant proteins.

Because the experiment did not include technical replicates, the models in *limma* and one-way ANOVA (implemented in *Proteome Discoverer*) were similar (but not identical) to the model in *MSstatsTMT* in supplemental Eq. S2. Fig. 6 and supplemental Fig. S5.3 illustrate that increasing the number of biological replicates (and the number of mixtures) improved the sensitivity and the specificity of most workflows. This underscored the importance of biological replicates for achieving accurate results.

At the same time, additional mixtures introduced more missing values, with up to 60% of proteins having at least one missing summary (supplemental Fig. S5.4). Therefore, the difference in performance was due primarily to the treatment of missing values. In *limma*, the negative impact of treatment of missing values and of the Empirical Bayes step was exacerbated and resulted in a large number of false positive differentially abundant proteins. One-way ANOVA was less sensitive than *MSstatsTMT*, however the discrepancy became smaller with the increase of sample size. *edgeR* filtered out proteins with missing summaries, and therefore reported the smallest number of testable proteins, true positive and false positive differentially abundant proteins.

Supplemental Fig. S5.5 further illustrates the differences between the workflows in the case of one example protein. In this example, the protein was entirely missing in 12 mixtures and therefore was not testable with *edgeR*. *limma* underestimated the standard error and identified the background protein as differentially abundant. Overall, *MSstatsTMT* reported more true positives while controlling the false discovery rate as the number of mixtures and percentage of missing values increased.

##### Evaluation on Biological Investigations with Diverse Designs

We evaluated *MSstatsTMT* in three biological investigations, each illustrating different challenges related to their experimental designs.

**Fig. 7:**
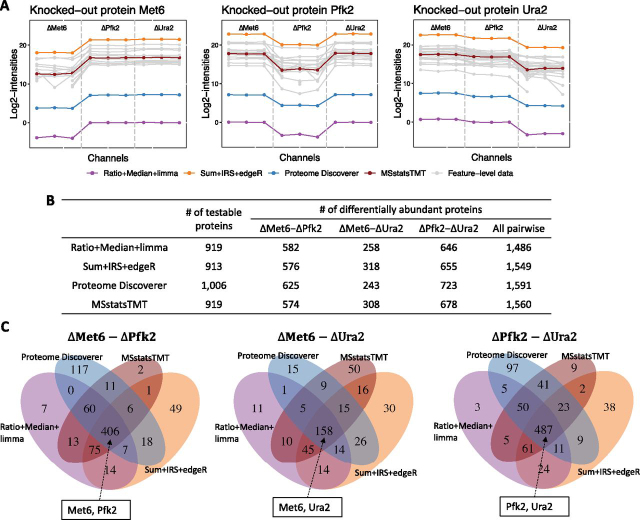
**Detection of differentially abundant proteins in all pairs of conditions in TKO-1mix (FDR cutoff of 0.05).***A*, Profile plot of the three knockout proteins Met6, Ura2, and Pfk2. Because the experiment consisted of one MS run, the plots only have one panel. Gray lines are the features of the protein. Color lines are *log*_2_ protein intensities summarized by the methods. To make the scale of the protein summaries comparable in the profile plot, the normalized protein intensities estimated by *Proteome Discoverer* and *Sum*+*IRS*+*edgeR* were *log*_2_ transformed. *B*, Number of testable proteins and number of differentially abundant proteins for each pairwise comparison. “All pairwise” indicates the total number of differentially abundant proteins across the three comparisons. *C*, Overlap of proteins detected as differentially abundant by *MSstatsTMT*, *Proteome Discoverer*, *Ratio*+*Median*+*Limma*, and *Sum*+*IRS*+*edgeR* for each pairwise comparison.

In the TKO-1mix investigation, all four workflows detected the knockout proteins as differentially abundant: The investigation profiled three knockout conditions, with three biological replicates each, in one MS run. This simple design produced few missing values, and *MSstatsTMT* fit a simple model in supplemental Eq. S4. As the result, all the workflows in Table I had a similar performance (Fig. 7). Normalized protein summaries reported by every workflow were similar, and lower in the corresponding knockout proteomes. All the workflows correctly detected the knockout proteins as differentially abundant. The workflows reported similar numbers of testable proteins, and a similar set of differentially abundant proteins. *Proteome Discoverer* included around 100 more “master” proteins from the ambiguous protein group, which were filtered out by *MSstatsTMT* converter prior to using the other workflows.

**Fig. 8:**
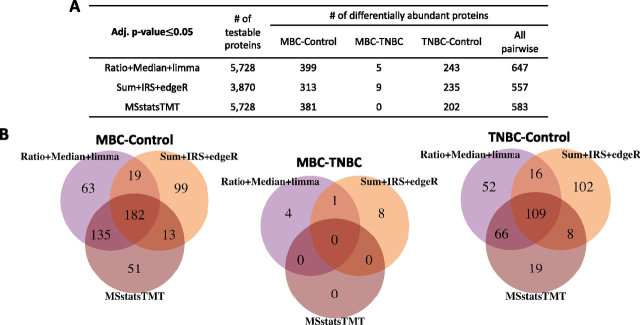
**Detection of differentially abundant proteins in all pairs of conditions in Human-3mix-balanced experiment (FDR cutoff of 0.05).***A*, Number of testable proteins and number of differentially abundant proteins for each pairwise comparison. “All pairwise” indicates the total number of differentially abundant proteins across the three comparisons. *B*, Overlap of proteins detected as differentially abundant by *MSstatsTMT*, *Proteome Discoverer*, *Ratio*+*Median*+*Limma*, and *Sum*+*IRS*+*edgeR* for each pairwise comparison.

In the Human-3mix-balanced investigation, differentially abundant proteins were dependent on the statistical model in each workflow: 27 human samples were profiled in three TMT 10-plex mixtures and no technical replicates. Nearly 33% of proteins had at least one missing summary. In this balanced design, all normalization and summarization methods produced relatively similar results. *MSstatsTMT* fit the model in supplemental Eq. S2. Fig. 8 summarizes the results.

*MSstatsTMT* and *Ratio*+*Median*+*Limma* reported the maximal number of testable proteins. *Ratio*+*Median*+*Limma* reported most differentially abundant proteins, and *MSstatsTMT* reported an intermediate number of differentially abundant proteins. This is consistent with the results on the controlled mixtures, where *Ratio*+*Median*+*Limma* underestimated the standard error of proteins with missing runs. *Sum*+*IRS*+*edgeR* required an additional internal normalization step to correct the protein summaries with respect to differences in “library size”. Although this normalization had little impact in the case of the controlled mixtures, it reduced the number of differentially abundant proteins from 1900 to 557 in this data set. Moreover, *Sum*+*IRS*+*edgeR* lost many testable proteins because of missing values and had the least reproducible results of tests for differential abundance. Only one protein out of the nine differentially abundant proteins in the comparison MBC-TNBC detected by *Sum*+*IRS*+*edgeR* was also found with another workflow.

**Fig. 9:**
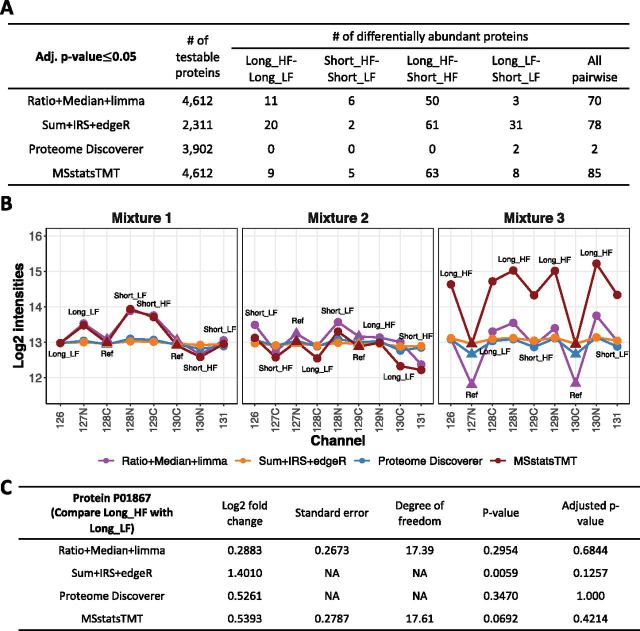
**Detection of differentially abundant proteins in all pairs of conditions in Mouse-3mix-unbalanced (FDR cutoff of 0.05).***A*, The number of testable proteins and the number of detected differentially abundant proteins. 'All pairwise' is the total number of differentially abundant proteins across comparisons. *B*, The normalized summaries of protein P01867 (Ig gamma-2B chain C region, IGG2B mouse). Each panel represents one MS run. *y*-axis: *log*_2_ intensity. *x*-axis: TMT channels. The labels inside each panel are the conditions in the corresponding channel and mixture. Color lines are *log*_2_ protein intensities summarized by each workflow. To make the scale of the protein summaries comparable in the profile plot, the normalized protein intensities estimated by *Proteome Discoverer* and *Sum*+*IRS*+*edgeR* were *log*_2_ transformed, and the protein summaries in Channel 126 of Mixture 1 were equalized across all the methods. *C*, Results of comparison between Long_HF and Long_LF for P01867.

In the Mouse-3mix-unbalanced investigation, both normalization and representation of sources of variation played an important role in detecting differentially abundant proteins: The investigation profiled 20 mouse samples within three TMT 10-plex mixtures in an unbalanced design. Nearly 54% of proteins had at least one missing summary across the three mixtures. *MSstatsTMT* fit the model in supplemental Eq. S2. Fig. 9*A* summarizes the results.

Consistent with the previous results, *MSstatsTMT* and *Ratio*+*Median*+*Limma* reported the maximal number of testable proteins, whereas *Sum*+*IRS*+*edgeR* lost testable proteins because of missing values. Unlike in the previous results, *MSstatsTMT* reported most differentially abundant proteins. This is due in part to the step of protein-level normalization. Fig. 9*B* and 9*C* illustrate in the example of one protein that in this unbalanced design the ratio normalization in *Ratio*+*Median*+*Limma* normalized away the fold changes between conditions. In particular, the protein summaries in reference channel of Mixture 3 shifted away from those in Mixture 1 and Mixture 2. This produced the smallest estimate of the absolute fold change. *Sum*+*IRS*+*edgeR* used the reference channel for normalization, but produced a large estimate of fold change, likely because of the Negative Binomial distribution assumption that can be negatively affected by outliers.

The statistical modeling in *Proteome Discoverer* did not consider the between-mixture effect and detected most proteins as nonsignificant. Overall, *MSstatsTMT* balanced the estimation of the fold change, standard error, and degrees of freedom for this unbalanced experimental design.

## DISCUSSION

This manuscript proposes a statistical workflow for detecting differentially abundant proteins in MS-based proteomic experiments with TMT labeling. The workflow is implemented as an open-source R/Bioconductor package, which takes as input exports from data processing tools such as Proteome Discoverer, MaxQuant, OpenMS, or SpectroMine.

Our evaluations indicate that performance of *MSstatsTMT*, as well as of all the other workflows, depends on the intrinsic characteristics of the investigation. For the protein-level normalization step, the use of a reference channel has little impact on the detection of differentially abundant proteins in balanced designs. However, in unbalanced designs, normalization without a reference channel can eliminate true fold changes, and reference channel-based normalization is preferred. The presence of a reference channel also improves the probability of selecting a peptide ion for fragmentation and the accuracy of the peptide identification. Therefore, *MSstatsTMT* encourages the users to add a reference channel to their designs.

Similarly, for the statistical modeling and inference step, most workflows performed similarly well in simple designs such as TKO-1mix. For the controlled mixtures with no biological variation, high signal-to-noise ratio, and balanced designs, *MSstatsTMT* performed similarly to *Ratio*+*Median*+*Limma* and *Proteome Discoverer*. The situation changes when the investigation incorporates larger biological variation, more biological replicates and mixtures, and combinations of biological and technical replicates. Such complex designs require more consideration regarding statistical modeling. Because *limma*, *edgeR* and one-way ANOVA do not distinguish between the biological and the technical variance and have limitations in handling missing values. This leads to inaccuracies in the inference, and loss of performance. In contrast, *MSstatsTMT* selects an appropriate model for each protein, and reflects both the experimental design and the pattern of missing protein summaries. This increases the sensitivity of detecting differentially abundant proteins while controlling false positive rate.

Beyond the performance of the workflows, experimentalists should always question the assumptions underlying each workflow. For example, the assumption of a Negative Binomial distribution in *edgeR*, which is inappropriate for TMT experiments, contributed to the negative performance of the workflow on the evaluation datasets. Similarly, global peptide normalization assumes that only a small proportion of proteins differ between the conditions. This assumption may not hold, *e.g.* in TKO-1mix where reporter ion intensities of peptides from ΔPfk2 were systemically below those from the other conditions. Athough this may reflect a technical artifact, it is also possible that the intervention affected the expression of many proteins.

Although statistical analyses in this manuscript-controlled FDR at 5%, the empirical FDR reported on the controlled mixtures was quite higher. A possible reason for this is spectral misidentification during the upstream steps of data processing. Intensity patters in the controlled mixtures indicated that some spectra from the spiked-in proteins were incorrectly identified as the background, thus inflating the empirical FDR. In biological investigations, FDR control can be similarly affected by spectral misidentifications.

Evaluations in this manuscript also assumed that the spectral features were mapped unambiguously to a protein sequence, and that the data set did not contain protein groups. Unlike the assumptions above, this assumption is not essential, and was only made for convenience of the evaluation. *MSstatsTMT* can also be applied to protein groups, in which case protein summarization and statistical modeling and inference will refer to representative patterns of the entire group. It may also be possible to rescue the shared peptides and further separate the protein groups, and this may be one direction of future research.

Overall, we hope that the statistical methodology and its implementation will be useful to many experimentalists relying on multiplexed MS-based proteomics in their work.

## DATA AVAILABILITY

All the datasets for the four experiments, from raw files to statistical analysis results, are available online.

The details of the experimental design, the raw data, and the intermediate data processing outputs are deposited in each MassIVE repository: SpikeIn-5mix-MS3 data (MassIVE repository identifier: MSV000084264, ProteomeXchange identifier: PXD0015258), SpikeIn-5mix-MS2 data (MassIVE repository identifier: MSV000084266, ProteomeXchange identifier: PXD015261), TKO-1mix data (MassIVE repository identifier: MSV000084263, ProteomeXchange identifier: PXD015257), Human-3mix-balanced (MassIVE repository identifier: MSV000085343, ProteomeXchange identifier: PXD014414), Mouse-3mix-unbalanced data (MassIVE repository identifier: MSV000082569, ProteomeXchange identifier: PXD005953)

The PSM quantification reports, the R scripts for statistical analysis, and the results for differential abundance analysis by each method are available in each MassIVE.quant reanalysis container. SpikeIn-5mix-MS3 data (MassIVE.quant reanalyses container identifier: RMSV000000265), SpikeIn-5mix-MS2 data (MassIVE.quant reanalyses container identifier: RMSV000000266), TKO-1mix data (MassIVE.quant reanalyses container identifier: RMSV000000267), Human-3mix-balanced (MassIVE.quant reanalyses container identifier: RMSV000000323), Mouse-3mix-unbalanced data (MassIVE.quant reanalyses container identifier: RMSV000000264).
