# Supplementary material for: MSstatsTMT: Statistical Detection of Differentially Abundant Proteins in Experiments with Isobaric Labeling and Multiple Mixtures
Source: Mol Cell Proteomics. 2020 Nov 25;19(10):1706–23. doi: 10.1074/mcp.RA120.002105 (PMC8015007; doi:10.1074/mcp.RA120.002105)
Supplement: Supplementary file 1 [file mmc1.pdf]

MSstatsTMT: Statistical detection of differentially abundant proteins in experiments with isobaric labeling and multiple mixtures

Supplementary information

Ting Huang[1,*], Meena Choi[1,*], Manuel Tzouros[2], Sabrina Golling[2], Nikhil Janak Pandya[2], Balazs Banfai[2], Tom Dunkley[2], Olga Vitek[1,$]

1 Khoury College of Computer Sciences, Northeastern University, Boston, MA, USA
2 Roche Pharma Research and Early Development, Pharmaceutical Sciences-BiOmics and Pathology, Roche Innovation Center Basel, Hoffmann-La Roche Ltd, Basel, Switzerland
∗ Authors contributed equally to the work

Contents

1 Experimental datasets **3**
- 1.1 Overview of data characteristics . . . 3
- 1.2 SpikeIn-5mix-MS3 and SpikeIn-5mix-MS2 (Five controlled mixtures with technical replicates) . . . 4
- 1.3 TKO-1mix (A triple knockout proteomics standard) . . . 7
- 1.4 Human-3mix-balanced (Breast cancer samples with fractionatiosn) . . . 7
- 1.5 Mouse-3mix-unbalanced (Mouse epididymal adipose tissue mixture with fractionation) . . . 7

2 Representative normalization and summarization workflows **9**

3 Statistical modeling and inference in MSstatsTMT **12**
- 3.1 A general linear mixed effects model for balanced split-split-plot designs . . . 12
- 3.2 MSstatsTMT: group comparison designs with biological variation, multiple mixtures, and multiple technical replicates . . . 14
- 3.3 MSstatsTMT: group comparison designs with biological variation, multiple mixtures, and single technical replicate . . . 15
- 3.4 MSstatsTMT: group comparison designs with biological variation, single mixture, and multiple technical replicates . . . 16
- 3.5 MSstatsTMT: group comparison designs with biological variation, single mixture, and single technical replicate . . . 17
- 3.6 MSstatsTMT: group comparison designs without biological variation (controlled mixtures) . . . 18
- 3.7 MSstatsTMT: Details of parameter estimation and testing for differential abundance . . . 19

4 Empirical details of model development in MSstatsTMT **20**
- 4.1 Mouse-3mix-unbalanced . . . 20
- 4.2 SpikeIn-5mix-MS3 . . . 21

5 Evaluation of performance : SpikeIn-5mix-MS3 and SpikeIn-5mix-MS2 experiment **22**

Section 1: Experimental datasets

1.1 Overview of data characteristics

Dataset	# of measurements	# of peptide ions	# of proteins	Mean # of peptide ions per protein group	# of differentially abundant proteins
SpikeIn-5mix-MS3	2,682,762	44,021	4,812	9.15	21
SpikeIn-5mix-MS2	3,365,207	55,519	5,519	10.06	20
TKO-1mix	37,754	4,203	919	4.57	>= 3
Human-3mix-unbalanced	1,267,457	65,076	5,763	11.29	Not available
Mouse-3mix-unbalanaced	643,853	39,752	4,713	8.43	Not available

Supplementary Figure S1.1: Characteristics of the experimental datasets in this manuscript For each dataset except BC-3mix-balanced, reporter ion intensities in PSM (peptide-to-spectrum matches) were obtained with Proteome Discoverer 2.2. For Human-3mix-balanced, reporter ion intensities were generated as the output files of Philosopher. The '# of measurements' column is the number of reporter ion intensities across all the channels of all the MS runs for all the PSMs. '# of peptide ions' is the number of peptide ions and '# of proteins' is the number of proteins (including protein groups with multiple proteins). 'Mean # of peptide ions per protein group' divides '# of peptide ions' by '# of proteins'. SpikeIn-5mix-MS3 and SpikeIn-5mix-MS2 has 28 truly differentially abundant USP1 proteins after excluding the ones confounded with the endogenous "light" species from SILAC-HeLa sample, and 21 truly differentially abundant USP1 proteins after further excluding the ambiguous protein groups (see Supplementary Section S1.2). In the TKO-1mix experiment three proteins were knocked out from the yeast proteome, and therefore has at least 3 differentially abundant proteins. Mouse-3mix-unbalanced is a biological investigation without ground-truth for differential abundance.

1.2 SpikeIn-5mix-MS3 and SpikeIn-5mix-MS2 (Five controlled mixtures with technical replicates)

Materials SILAC K8/R10 HeLa cell pellet was from Dundee Cell Products (Dundee, UK). TMT10-plex isobaric label reagents were from Thermo Fisher Scientific (Rockford, IL, USA). Universal Proteomics Standard Set (UPS1) and all other chemicals were from Sigma-Aldrich (Steinheim, Germany) unless otherwise stated.

Protein digestion and controlled mixture preparation Heavy SILAC HeLa cell pellet was homogenized in lysis buffer (1% SDS, 8 M urea, 50 mM TEAB pH 8.5, protease inhibitors (cOmplete, Roche) at RT at a concentration of 2.5E4 cells/μl using a tip sonifier (QSonica, Newtown, CT), applying 3×10 s bursts (30% amplitude) and cooling the samples between bursts on ice for 1 min. Protein concentration was determined using the bicinchoninic assay (BCA, Pierce). Proteins were precipitated using methanol/chloroform. Briefly, four, one, and three volumes of methanol, chloroform, and water, respectively, were added to the lysate followed by vortexing after each solvent addition, and final centrifugation at 15'000 $\times$ g at RT for 10 min. After removal of the supernatant, protein pellet was washed twice with cold methanol and air dried. Heavy SILAC HeLa protein pellet and 48 UPS1 proteins were separately re-dissolved in digestion buffer (8 M urea, 50 mM TEAB pH 8.5) at a concentration of 5 and 0.2 μg/μl, respectively, reduced with 10 mM (end concentration) DTT at 56 °C for 30 min and alkylated with 20 mM (end concentration) iodoacetamide at RT for 30 min (dark). SILAC HeLa and UPS1 proteins were pre-digested with 1:100 and 1:20 w/w lysyl endopeptidase (Lys-C, Wako Pure Chemical Industries Ltd., Japan), respectively, at 37 °C for 4 h. Samples' urea concentration was diluted to 2 M with 50 mM TEAB pH 8.5 solution, and digestion was continued by the addition of 1:100 and 1:20 w/w trypsin (Promega, Madison, WI), respectively, and incubation at 37 °C overnight. Samples were acidified with FA to a final 5% v/v content, vortexed, and centrifuged at 7'000 $\times$ g at 4 °C for 10 min to remove insoluble material. Heavy SILAC HeLa and UPS1 peptides were desalted using solid-phase extraction (SPE) (50 mg C18 Sep-Pak, Waters, Milford, MA) and homemade StAGE tips (prepared with C18 material from 3M Empore) [1], respectively, and eluates used to prepare controlled mixtures. 500, 333, 250, and 62.5 fmol UPS1 peptides were combined with 50 μg SILAC HeLa peptides in duplicate in order to generate a dilution series corresponding to 2/3, 1/2, and 1/8 of the highest UPS1 peptide amount (500 fmol). In addition, a reference sample was generated by pooling all four diluted UPS1 peptide samples (286.5 fmol) and combined with 50 μg of SILAC HeLa in duplicate. A total of five controlled mixtures were prepared and all samples were dried down using a speedvac. The overall experimental design is shown in Supplementary Figure S1.2.

Peptide Tandem Mass Tag labelling TMT labeling was performed according to Paulo *et al.*[2] with some minor modifications. Dried peptides were reconstituted in 100 mM HEPES pH 8.5 buffer (ca. 0.7 μg/μl final concentration) and individually labeled with 150 μg TMT10-plex reagents (dissolved in anhydrous ACN, 30% v/v end concentration) at RT for 85 min.

The reaction mixtures were quenched with hydroxylamine (0.3% v/v end concentration) at RT for 20 min followed by acidification with FA (5% v/v end concentration). Samples were combined within their respective controlled mixtures 1-5, evaporated to dryness (speedvac), desalted by SPE (50 mg C18 Sep-Pak), dried down and stored at -20 °C until analysis.

LC-MS/MS LC-MS/MS was performed using an EASY-nLC 1200 ultrahigh pressure liquid chromatography (UHPLC) connected to an Orbitrap Fusion Lumos Tribrid and equipped with an EASY-spray source (Thermo Fisher Scientific, San Jose, CA). Samples were re-suspended in 5% FA/2% ACN, and concentrated on an Acclaim PepMap C18 trapping column (75 μm $\times$ 20 mm, 5 μm particle size) at a controlled maximum backpressure of 500 bar. Approximately 2 μg sample (SILAC HeLa peptides) containing 2, 1.333, 1, 0.25, and 1.146 fmol (5.729 fmol in duplicate, ca. 11.4 fmol total) UPS1 peptides corresponding to the 1, 2/3, 1/2, 1/8, and reference samples dilutions was loaded and run in triplicate for each controlled mixture. Peptides were separated on an Acclaim PepMap C18 EASY-spray column (75 μm $\times$ 750 mm, 2 μm particle size) heated at 45 °C and using the following gradient at 270 nl/min: 5% B for 5 min, 5-20% B in 120 min, 20-45% B in 120 min, 45-100% B in 5 min, 100% B for 20 min, corresponding to a total acquisition time of 270 min (buffer A: 0.1% FA; buffer B: 0.1% FA/80% ACN). The spray voltage used was 1.9-2.2 kV.

The SpikeIn-5mix-MS3 data were acquired using an MS2/MS3 (also called "multinotch MS3" or Synchronous

TMT10plex reagent		126	127N	127C	128N	128C	129N	129C	130N	130C	131
Mixture 1	Run 1	Ref	0.667	0.125	0.5	1	0.125	0.5	1	0.667	Ref
	Run 2	Ref	0.667	0.125	0.5	1	0.125	0.5	1	0.667	Ref
	Run 3	Ref	0.667	0.125	0.5	1	0.125	0.5	1	0.667	Ref
Mixture 2	Run 4	Ref	0.5	1	0.667	0.125	1	0.667	0.125	0.5	Ref
	Run 5	Ref	0.5	1	0.667	0.125	1	0.667	0.125	0.5	Ref
	Run 6	Ref	0.5	1	0.667	0.125	1	0.667	0.125	0.5	Ref
Mixture 3	Run 7	Ref	0.125	0.667	1	0.5	0.5	0.125	0.667	1	Ref
	Run 8	Ref	0.125	0.667	1	0.5	0.5	0.125	0.667	1	Ref
	Run 9	Ref	0.125	0.667	1	0.5	0.5	0.125	0.667	1	Ref
Mixture 4	Run 10	Ref	1	0.5	0.125	0.667	0.667	1	0.5	0.125	Ref
	Run 11	Ref	1	0.5	0.125	0.667	0.667	1	0.5	0.125	Ref
	Run 12	Ref	1	0.5	0.125	0.667	0.667	1	0.5	0.125	Ref
Mixture 5	Run 13	Ref	0.667	0.125	0.5	1	0.125	0.5	1	0.667	Ref
	Run 14	Ref	0.667	0.125	0.5	1	0.125	0.5	1	0.667	Ref
	Run 15	Ref	0.667	0.125	0.5	1	0.125	0.5	1	0.667	Ref

Supplementary Figure S1.2: Design of SpikeIn-5mix-MS3 and SpikeIn-5mix-MS2 Each row is a mixture, profiled in three technical replicate MS runs. Each column is a channel. Colors show conditions that represent the concentration of UPS1 proteins. The last column represents the reference channel and is used for local protein normalization. Each entry is the TMT10-plex label of a sample. Mixture 1 and 5 are identical since they have exactly the same design.

Precursor Selection, SPS) [3]. The SpikeIn-5mix-MS2 data were acquired using MS2-only strategies. For the MS2/MS3, the instrument was operated in the data-dependent acquisition (DDA) mode, collecting Orbitrap full MS1 scans over a mass range from m/z 300 to 1400 using quadrupole isolation, a resolution of 120k (at m/z 200), an automatic gain control (AGC) target value of 2E5, and a maximum injection time (IT) of 50 ms. Data were on-the-fly recalibrated using ambient air hexacyclodimethylsiloxane at m/z 445.12002. During a cycle time of 3 sec (top speed), the most intense precursor ions, with charge states between 2 and 6, a minimum intensity of 5E3, were mono-isotopically selected for collision induced dissociation (CID), using a quadrupole isolation of m/z 0.7, AGC target of 1E4, maximum IT of 50 ms, collision energy of 35%, and ion trap readout with turbo scan rate. Only a single charge state per precursor was selected for MS2. Interrogated precursor ions were dynamically excluded for 75s using a $\pm$10 ppm mass tolerance. TMT reporter ions were generated using SPS, a quadrupole isolation of m/z 2, high-energy collision dissociation (HCD) at a normalized collision energy of 65%, and readout in the Orbitrap with a resolution of 60k, scan range of m/z 100 to 500, an AGC target of 5E4, and a maximum IT of 105 ms. The mass range for selecting the SPS (MS3) precursors was from m/z 400 to 2000, excluding the MS2 precursor with a tolerance of m/z 40 (low) and 5 (high), and any TMT neutral loss from it. The number of SPS precursors was set to 10. Alternatively, the instrument was operated in MS2-only mode using HCD at a normalized collision energy of 40%, AGC target of 5E4, and a maximum IT of 115 ms. All the other relevant parameters were essentially the same as above.

Data processing Raw data of SpikeIn-5mix-MS3 and SpikeIn-5mix-MS2 were processed using Proteome Discoverer 2.2.0.388 (Thermo Fisher Scientific) and Mascot Server 2.6.1 (Matrix Science, London). Processing workflows were designed to perform a two-pass search on the MS2 data for SpikeIn-5mix-MS3 and SpikeIn-5mix-MS2. First, the data were searched against the UniProt/SwissProt human protein database (07.2018 release, 20'398 entries) using trypsin/P as an enzyme, a maximum of two missed cleavage sites, and 10 ppm, as the precursor ion mass tolerance. 0.5 and 0.1 Da were specified as the fragment ion mass tolerances for SpikeIn-5mix-MS3 and SpikeIn-5mix-MS2, respectively. Carbamidomethylated cysteines (+57.02146 Da), TMT10 and SILAC K8 labeled lysines (+237.177131 Da), SILAC

R10 arginines (+10.008269 Da), and TMT10 labeled peptide N-termini (+229.162932 Da) were set as static, while oxidized methionines (+15.99492 Da) were set as dynamic modifications. Second, the data were searched against the human protein database for which the endogenous sequence were replaced with UPS sequences (protein entries marked with _ups, 20'395 entries), using carbamidomethylated cysteines, TMT10 labeled lysines and peptide N-termini as static, and oxidized methionines as dynamic modifications. The peptide-to-spectrum matches (PSMs) false discovery rates (FDRs) were controlled using Percolator and setting a max. delta Cn of 0.05 and a q-value threshold of 0.01. Reporter ion quantitation was performed using the MS2, and MS3 data order for SpikeIn-5mix-MS3 and SpikeIn-5mix-MS2, 3 mmu peak integration and most confident centroid tolerances. Reporter ion intensities were adjusted to correct for the isotopic impurities of the different TMT reagents (manufacturer specifications). Reporter ions intensities were used to express abundances.

PSMs from all ranks were considered, peptide FDRs were controlled by setting a q-value threshold of 0.01 and allowing the software to automatically select PSM q-value or ion score for the grouping (PSM FDR was 0.89%, peptide group FDR was 1.21%). High confidence peptides with a minimal length of 6 residues were further grouped into proteins and protein FDR was set to fulfill a q-value threshold of 0.01. At this level, protein FDR was 1.09%. For protein grouping, strict parsimony principle was applied.

We exported the PSM report from Proteome Discoverer to R and used 'PDtoMSstatsTMTFormat()' from *MSstatsTMT* package to perform filtering on reporter ion intensities. The PSM report contained 6767 proteins for the MS2 and 5903 proteins for the MS3 data. The filters includes removing shared peptides and peptides with one or two reporter ion intensities within each MS run. The function also summarized spectra intensities to peptide ion intensities by selecting a "representative" spectrum with the minimal number of missing reporter ion intensities, highest reporter ion intensity, or lowest interference score if the information was available. 1,000 and 1,203 ambiguous protein groups which contained multiple proteins were filtered out from SpikeIn-5mix-MS3 and SpikeIn-5mix-MS2, respectively. We removed the ambiguous proteins since we would like to make statistical conclusion for each single protein, not the protein group. In particular, protein groups may contain both spiked-in proteins and background proteins. Therefore, we could not determine the true fold changes for these groups, and omitted these groups from the analysis. 40 UPS1 proteins were remaining after the removal of the ambiguous proteins for both SpikeIn-5mix-MS3 and SpikeIn-5mix-MS2. For SpikeIn-5mix-MS3, the downstream statistical analysis were performed on 21 out of the 40 UPS1 proteins since the other 19 proteins were excluded as endogenous "light" species from SILAC-HeLa sample (see **Determination of endogenous UPS proteins in HeLa** section). Similarly, SpikeIn-5mix-MS2 had 20 UPS1 proteins. The final dataset consisted of 5520 proteins in the MS2 dataset, and 4865 proteins in the MS3 dataset.

Determination of endogenous UPS proteins in HeLa for SpikeIn-5mix-MS3 In order to identify which UPS1 proteins were also present as endogenous "light" species in the SILAC-HeLa sample, we analyzed separately aliquots of the SILAC-HeLa (2 ug), UPS1 (11.4 fmol), and UPS1 spiked-in SILAC-HeLa (11.4 fmol-2 ug, all without TMT labeling). Each sample was run in triplicate on an EASY-nLC 1000 connected to an Orbitrap Fusion Tribrid using the following adapted parameters. After loading on the trapping column, peptides were separated on a Acclaim PepMap C18 EASY-spray column (75 um x 500 mm) using a shorter gradient at 300 nl/min: 5% B for 5 min, 5-15% B in 60 min, 15-35% B in 60 min, 35-80% B in 2 min, 80% B for 18 min, corresponding to a total acquisition time of 140 min (buffer A: 0.1% FA; buffer B: 0.1% FA/ACN). Data were acquired using DDA, collecting Orbitrap full MS1 scans over a mass ranger from m/z 300 to 1500 and a maximum IT of 100 ms. Precursor ions were accumulated for a maximum IT of 35 ms, fragmented by HCD at a collision energy of 30%, and readout in the ion trap with rapid scan rate. Interrogated precursor ions were dynamically excluded for 20s. Raw data were processed with MaxQuant 1.5.3.30 and searched against a combined human (06.2016 release) and UPS protein database (20'249 total entries). Default parameters were used with the following modifications: trypsin/P was used as enzyme and maximum number of missed cleavages were set to 2; precursor mass tolerance was set to 20 and 4.5 ppm for the first and main searches, respectively; oxidized methionines, acetylated protein N-termini were set as variable, while carbamidomethylated cysteines were set as fixed modifications; PSM and protein false discovery rate was set to 1%; SILAC K8 and R10 were selected as labeled amino acids and unique peptides only were selected for quantification with a minimum ratio count of 2. As a result, 19 UPS1 proteins were identified and quantified in SILAC-HeLa, meaning 21 final candidates were kept for further analysis (i.e. # of spike-in proteins shown in Supplementary Figure S1.1)

1.3 TKO-1mix (A triple knockout proteomics standard)

The data was published in Paulo *et al.*[4]. The raw file was a generous gift from Joao Paolo (Harvard Medical School). The sample corresponds to a 1 ug yeast triple knock-out (TKO) sample on-column injection on an Orbitrap Fusion as described [4]. The reporter ion isotopic distributions of the TMT10-plex label reagent set was from lot number RK239786. The TMT channel to sample description is provided in Supplementary Figure S1.3.

TMT6plex reagent	126	127N	127C	128N	128C	129N	129C	130N	130C
Mixture 1	ΔMet6	ΔMet6	ΔMet6	ΔPfk2	ΔPfk2	ΔPfk2	ΔUra2	ΔUra2	ΔUra2

Supplementary Figure S1.3: Design of TKO-1mix Each row is a mixture. Each column is a channel. Colors show yeast deletion strains, labeled and profiled in triplicates.

Raw data were processed as described in SpikeIn-5mix-MS3 with some adaptations. A processing workflow was designed to search the MS2 data against the UniProt/SwissProt S. cerevisiae protein database (strain S288c, 07.2017 release, 9’721 entries). Carbamidomethylated cysteines (+57.02146 Da), TMT10 labeled lysines and peptide N-termini (+229.162932 Da) were set as static, while oxidized methionines (+15.99492 Da) were set as dynamic modifications. For this dataset, PSM FDR was 1.0%, peptide group FDR was 1.8% and protein FDR was 1.0%. The PSM report from Proteome Discoverer was then exported to R and same preprocessing steps were performed as in SpikeIn-5mix-MS3. 147 ambiguous protein groups which contained multiple proteins were filtered out.

1.4 Human-3mix-balanced (Breast cancer samples with fractionatiosn)

The biological study was published in Djomehri *et al.*[5] and the experimental design is described in Supplementary Figure S1.4. 15 MBC (Metaplastic breast carcinoma) samples, 6 TNBC (triple-negative breast cancer) samples, and 6 normal adjacent breast (Control) samples were equally allocated into three TMT 10-plex mixtures. Each mixture had one reference channel with a pool of all 27 samples (Supplementary Section S1.4). Each TMT mixture was separated into eight fractions to increase proteome coverage. The mixtures were profiled using SPS, producing a total of 24 MS runs. Raw data were analyzed by using MSFragger (v20181128) and Philosopher toolkit (v20181128, `github.com/Nesvilab/philosopher`). Three PSM tables from each TMT 10-plex experiment generated by Philosopher were obtained from the authors of the original paper. The PSM files was then exported to R and same preprocessing steps were performed as in SpikeIn-5mix-MS3. Additionally, multiple fractions belonging to same mixture were combined. If a peptide ion was shared by multiple fractions, we kept the fraction with maximal average reporter ion abundance across all the channel in the fraction.

TMT10plex reagent	126	127N	127C	128N	128C	129N	129C	130N	130C	131
Mixture 1	MBC	MBC	MBC	MBC	MBC	TNBC	TNBC	Control	Control	Ref
Mixture 2	MBC	MBC	MBC	MBC	MBC	TNBC	TNBC	Control	Control	Ref
Mixture 3	MBC	MBC	MBC	MBC	MBC	TNBC	TNBC	Control	Control	Ref

Supplementary Figure S1.4: Design of BC-3mix-balanced Each row is a mixture and each column is a channel. Color shows conditions. Each entry is the TMT10-plex label of a biological MBC (Metaplastic breast carcinoma), TNBC (triple-negative breast cancer), and normal breast (Control) sample from patients. Each mixture has an equal number of samples from each condition (i.e., has a balanced design) and is separated into eight fractions.

1.5 Mouse-3mix-unbalanced (Mouse epididymal adipose tissue mixture with fractionation)

The biological study was published in Plubell *et al.*[6] and the experimental design is described in Supplementary Figure S1.5. Raw files (27 files) were all downloaded from the PRIDE partner repository of the ProteomeXchange

Consortium found in the PXD005953 dataset identifier. The reporter ion isotopic distributions of the TMT10-plex label reagent set was from lot number QD212963.

TMT10plex reagent	126	127N	127C	128N	128C	129N	129C	130N	130C	131
**Mixture 1**	Long_LF	Long_LF		Short_LF	Ref		Short_HF	Short_HF	Ref	Short_LF
**Mixture 2**	Short_LF	Ref	Short_HF	Short_LF	Long_LF	Long_HF	Ref		Long_LF	Short_HF
**Mixture 3**	Long_HF	Ref		Long_HF	Long_LF	Long_HF	Short_HF	Long_HF	Ref	Short_LF

Supplementary Figure S1.5: Design of Mouse-3mix-unbalanced Each row is a mixture and each column is a channel. Color shows conditions. Each entry is the TMT10-plex label of a biological mouse sample. Each mixture has an unequal number of samples from each condition (i.e., has an unbalanced design) and is separated into nine fractions.

Raw data were processed as described in SpikeIn-5mix-MS3 with some adaptations. A processing workflow was designed to search the MS2 data against the UniProt/SwissProt mouse protein database (07.2017 release, 16909 entries). Carbamidomethylated cysteines (+57.02146 Da), TMT10 labeled lysines and peptide N-termini (+229.162932 Da) were set as static, while oxidized methionines (+15.99492 Da) were set as dynamic modifications. For this dataset, PSM FDR was 1.0%, peptide group FDR was 1.8% and protein FDR was 1.0%. The PSM report from Proteome Discoverer was then exported to R and same preprocessing steps were performed as in SpikeIn-5mix-MS3. Additionally, multiple fractions belonging to same mixture were combined. If a peptide ion was shared by multiple fractions, we kept the fraction with maximal average reporter ion abundance across all the channel in the fraction. 730 ambiguous protein groups which contain multiple proteins were filtered out.

Section 2: Representative normalization and summarization workflows

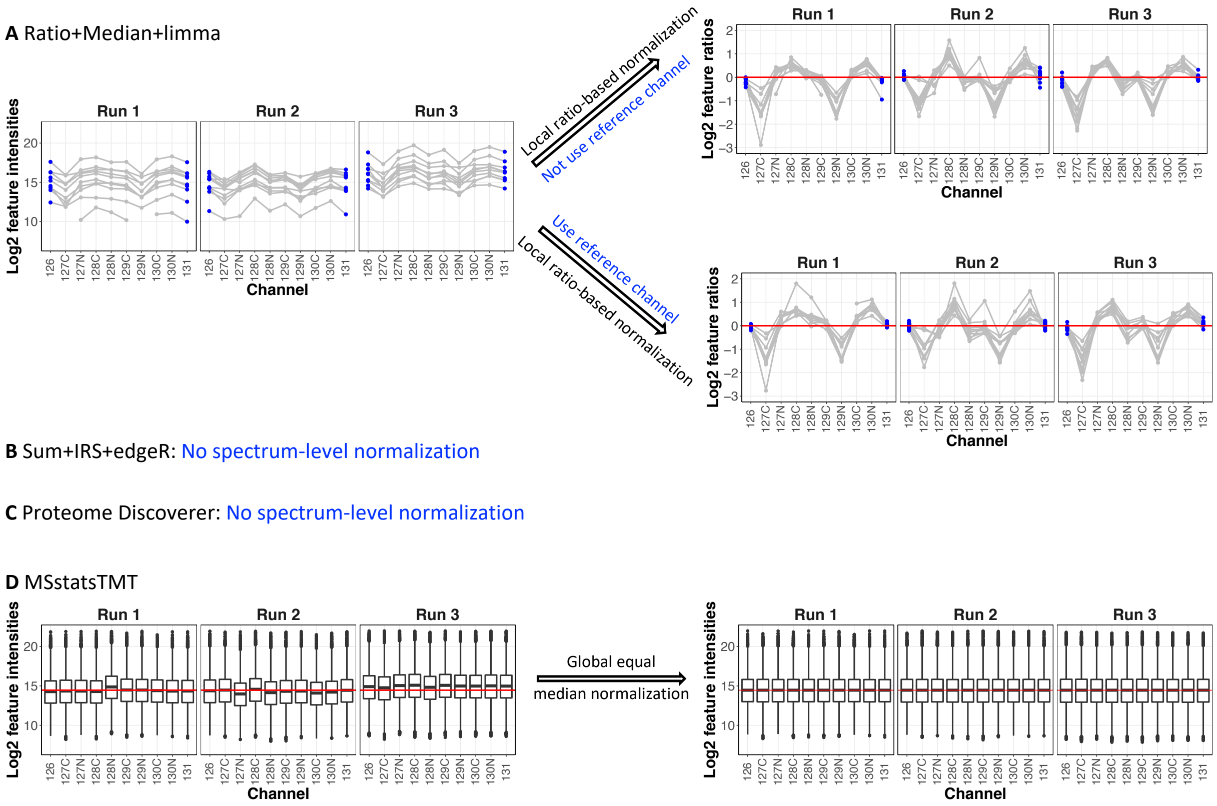

Supplementary Figure S2.1: Spectrum-level normalization in representative workflows in Table 1. The figure illustrates one protein in a hypothetical experiment with a reference channel and three runs. (A) Spectrum-level ratio normalization of *Ratio+Median+Limma*. Y-axis is on the log_2 intensity scale before normalization, and on the log_2 ratio scale after normalization. Gray lines are the features of the protein; blue dots indicate reference channels; red horizontal line indicates zero. Normalization with respect to the reference channel brings the log_2 feature intensities in the reference channel closer to zero than normalization without the reference channel. (B) *Sum+IRS+edgeR*: no spectrum-level normalization. (C) Proteome Discoverer: no spectrum-level normalization. (D) Global equal median normalization by *MSstatsTMT*. The normalization equalizes the median $loog_2$ intensities across all the channels and runs.

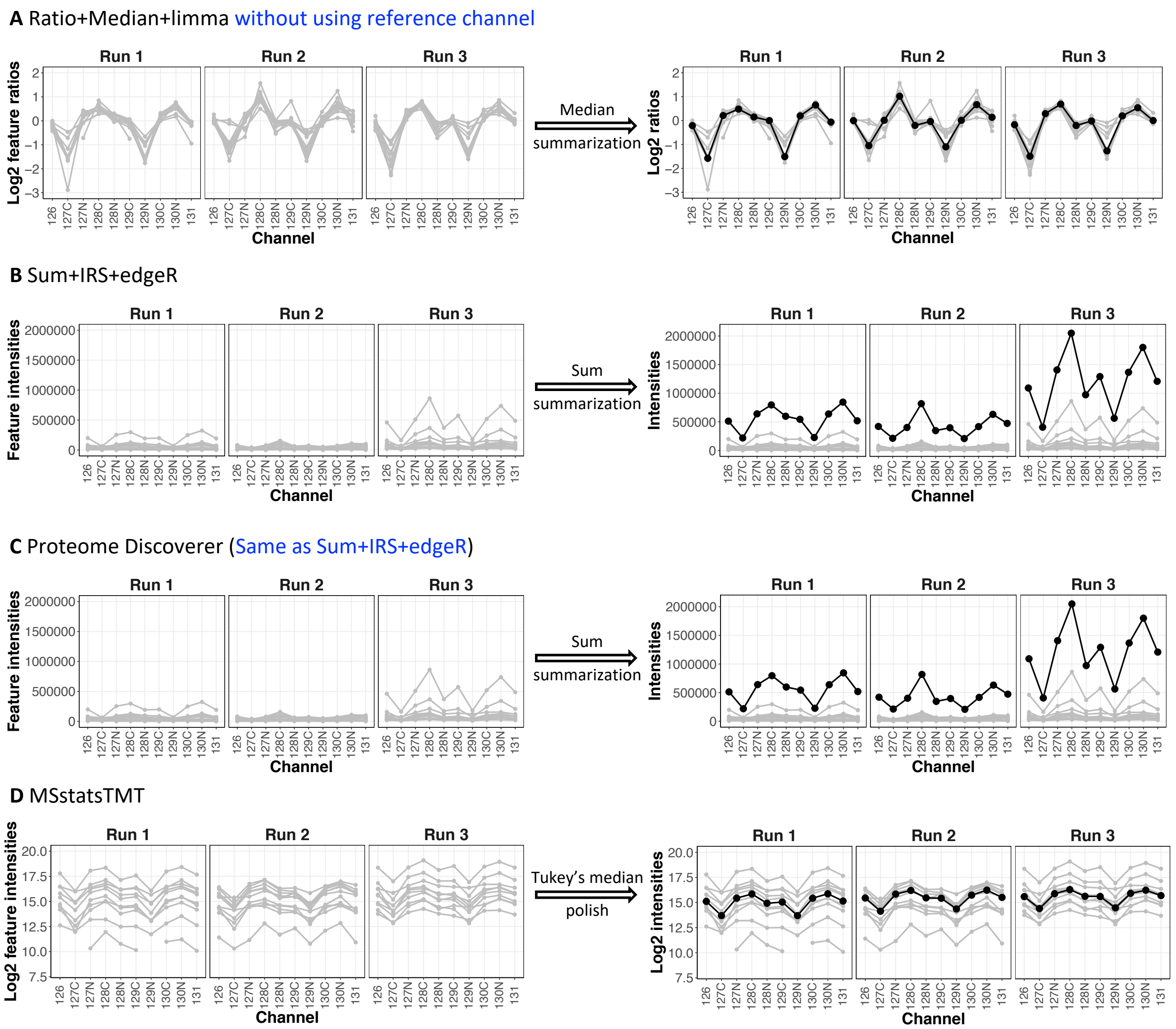

Supplementary Figure S2.2: Protein summarization in representative workflows in Table 1. The figure illustrates one protein in a hypothetical experiment with two reference channels and three runs. Gray lines are features of the protein, normalized on the spectrum level as in Supplementary Figure S2.1; black lines are protein summaries. (A) *Ratio+Median+Limma* summarizes log_2 feature ratios into log_2 protein ratios by calculating medians in each channel and run. (B) *Sum+IRS+edgeR* and (C) Proteome Discoverer sums all the feature intensities in each channel and run. (D) *MSstatsTMT* imputes missing values using Accelerated Failure Time Model, and summarizes log_2 feature intensities into log_2 protein summaries using Tukey's median polish.

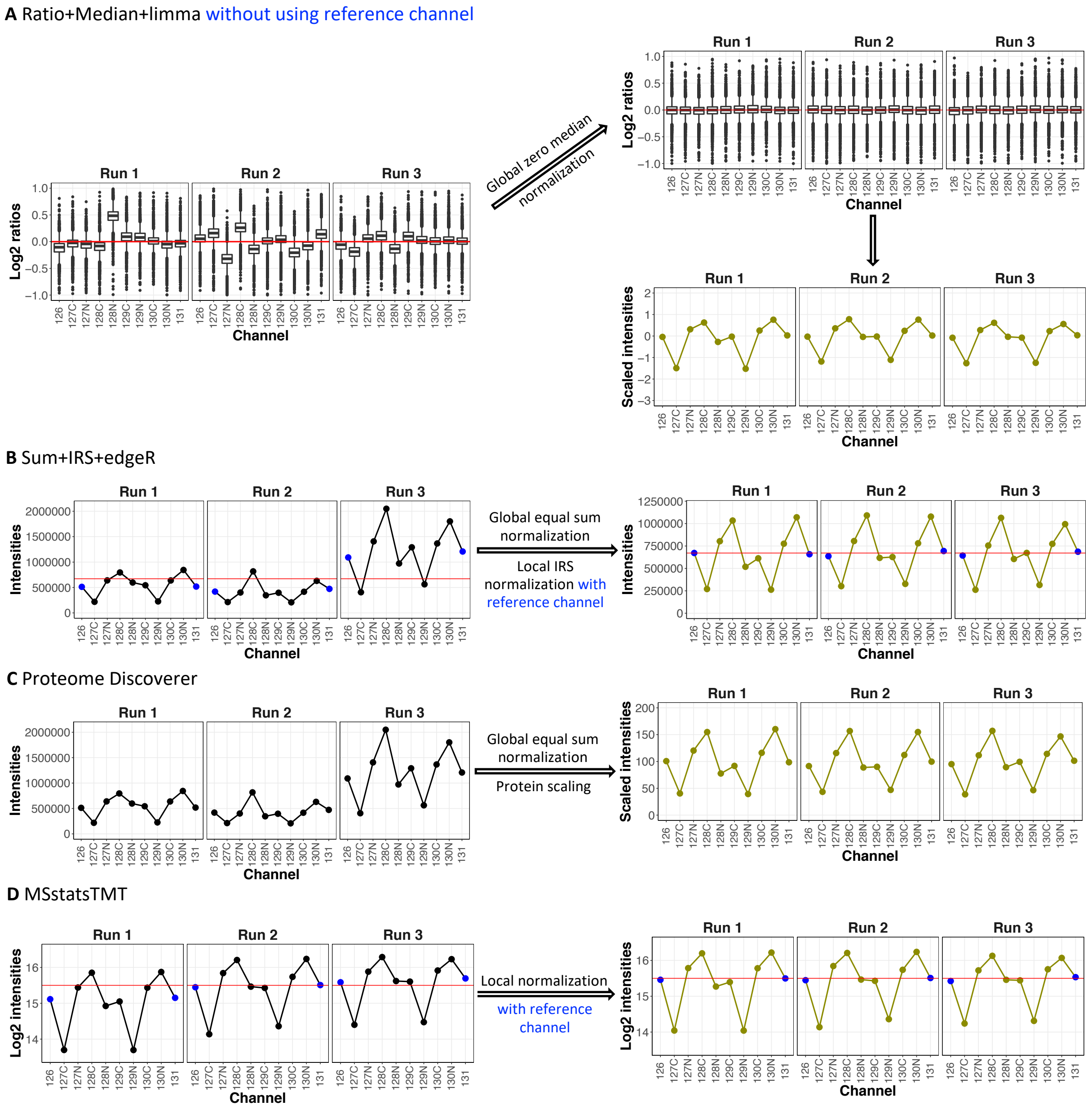

Supplementary Figure S2.3: Protein-level normalization in representative workflows in Table 1. The figure illustrates one protein in a hypothetical experiment with two reference channels and three runs. Blue dots indicate reference channels; black lines are protein summaries from Supplementary Figure S2.2. Yellow lines are normalized protein summaries. (A) For each run, the global zero median normalization by *Ratio+Median+Limma* shifts the median of log_2 protein ratio in each channel towards zero (red horizontal line). (B) *Sum+IRS+edgeR*, IRS normalization equalizes the normalized protein summaries in the reference channels across all the runs to the geometric mean (red horizontal line). (C) For each run, Proteome Discoverer equalizes the sum of the protein summaries across all the channels and then scales the equalized summaries of each protein to an average of 100. (D) *MSstatsTMT* equalizes the log_2 protein summaries in the reference channel across all the runs to the median (red horizontal line).

Section 3: Statistical modeling and inference in MSstatsTMT

3.1 A general linear mixed effects model for balanced split-split-plot designs

$$
\begin{aligned}
X_{mtcbf} = \mu &+ \mathbf{Mixture_m} + \mathbf{TechRep(Mixture)_{t(m)}} \\
&+ \mathbf{Condition_c} + \mathbf{Condition \times Mixture_{cm}} + \mathbf{Condition \times TechRep(Mixture)_{ct(m)}} \\
&+ \mathbf{BioRep(Mixture \times Condition)_{b(mc)}} + \mathbf{BioRep \times TechRep(Mixture \times Condition)_{bt(mc)}} \\
&+ \mathbf{Feature_f} + \epsilon_{\mathbf{mtcbf}}
\end{aligned}
$$

where $\mathbf{Mixture_m} \overset{iid}{\sim} N(0, \sigma^2_M)$, $\mathbf{TechRep(Mixture)_{t(m)}} \overset{iid}{\sim} N(0, \sigma^2_T)$

$\sum_{\mathbf{c=1}}^{\mathbf{C}} \mathbf{Condition_c} = 0$, $\mathbf{Condition \times Mixture_{cm}} \overset{iid}{\sim} N(0, \sigma^2_{CM})$

$\mathbf{Condition \times TechRep(Mixture)_{ct(m)}} \overset{iid}{\sim} N(0, \sigma^2_{CT})$

$\mathbf{BioRep(Mixture \times Condition)_{b(mc)}} \overset{iid}{\sim} N(0, \sigma^2_B)$

$\mathbf{BioRep \times TechRep(Mixture \times Condition)_{bt(mc)}} \overset{iid}{\sim} N(0, \sigma^2_{BT})$

$\sum_{\mathbf{f=1}}^{\mathbf{F}} \mathbf{Feature_f} = 0$, $\epsilon_{\mathbf{mtcbf}} \overset{iid}{\sim} \mathcal{N}(0, \sigma^2)$

Supplementary Figure S3.1: Linear mixed effects model for the split-split-plot design in MS experiment with isobaric labeling, with a group comparison design. Blue: whole plot; orange: subplot; pink: sub-subplot

The terms in the first line (in blue) describe the components of variation in the whole plot. The notation in parenthesis indicates that $TechRep$ is nested within $Mixture$ and the term $TechRep(Mixture)$ is the whole plot error. The terms in the second and third lines (in orange) describe the components of variation in the subplot. The notation in parenthesis indicates that $BioRep$ and $BioRep \times TechRep$ are nested within $Mixture \times Condition$ and the term $BioRep \times TechRep(Mixture \times Condition)$ is the subplot error. The terms in the fourth line (in purple) describe the components of variation in the sub-subplot and ϵ indicates the sub-subplot error. The sub-subplot error ϵ combines the contributions of $Feature \times Mixture$, $Feature \times TechRep(Mixture)$, $Feature \times Condition$, $Feature \times Condition \times Mixture$, $Feature \times Condition \times TechRep(Mixture)$, $Feature \times BioRep(Mixture \times Condition)$, $Feature \times BioRep \times TechRep(Mixture \times Condition)$. The constraints distinguish the fixed and the random components of variation. Comparisons of interest (i.e., conditions), are at the level of the subplot. Their standard errors are derived from the subplot components of variation.

Justification of the approach in MSstatsTMT: Supplementary Figure S3.2 shows the Analysis of Variance (ANOVA) decomposition for this model, in the case of a balanced design. The rows in the figure are the terms in Supplementary Figure S3.1. The columns summarize the properties of the model terms, and their estimation in the balanced case. As can be seen, all the terms in the whole plot (blue part of the figure) and in the subplot (orange part of the figure) do not depend on the observed log_2-intensities x_{mtcbf}, but only on their averages $\bar{x}_{mtcb.}$ over the features. In other words, testing for differential abundance between conditions in balanced designs only relies on the summaries of the reporter ion intensities over all the features in one channel and run. Therefore, conclusions from the general linear model are equivalent to the conclusions of a two-step procedure, which (1) summarizes the reporter ion intensities over all the features in one channel and run, and (2) fits a reduced linear model that expresses the whole plot and the subplot aspects of the design. In a special case of a balanced design with no missing feature intensities, the conclusions of *MSstatsTMT* are identical to the conclusions from the model in Supplementary Figure S3.3.

	Source of variation	Effect	Sum of Squares	Degrees of freedom	Expected Mean Square
Whole plot	Mixture	random	$TCBF\sum_{m=1}^{M}(\bar{x}_{m....}-\bar{x}_{.....})^2$	$M-1$	$\sigma^2+F\sigma_{BT}^2+TF\sigma_B^2+CBF\sigma_T^2+TCBF\sigma_M^2$
	TechRep(Mixture) (Whole plot error)	random	$CBF\sum_{m=1}^{M}\sum_{t=1}^{T}(\bar{x}_{mt...}-\bar{x}_{m....})^2$	$M(T-1)$	$\sigma^2+F\sigma_{BT}^2+CBF\sigma_T^2$
Subplot	Condition	fixed	$MTBF\sum_{c=1}^{C}(\bar{x}_{..c..}-\bar{x}_{.....})^2$	$C-1$	$\sigma^2+F\sigma_{BT}^2+TF\sigma_B^2+BF\sigma_{CT}^2+TBF\sigma_{CM}^2+\frac{MTBF\sum_j C_j^2}{C-1}$
	Condition × Mixture	random	$TBF\sum_{c=1}^{C}\sum_{m=1}^{M}(\bar{x}_{m.c..}-\bar{x}_{m....}-\bar{x}_{..c..}+\bar{x}_{.....})^2$	$(C-1)(M-1)$	$\sigma^2+F\sigma_{BT}^2+TF\sigma_B^2+BF\sigma_{CT}^2+TBF\sigma_{CM}^2$
	Condition × TechRep(Mixture)	random	$BF\sum_{m=1}^{M}\sum_{t=1}^{T}\sum_{c=1}^{C}(\bar{x}_{mtc..}-\bar{x}_{mt...}-\bar{x}_{m.c..}+\bar{x}_{m....})^2$	$M(C-1)$ $(T-1)$	$\sigma^2+F\sigma_{BT}^2+BF\sigma_{CT}^2$
	BioRep(Condition × Mixture)	random	$TF\sum_{m=1}^{M}\sum_{c=1}^{C}\sum_{b=1}^{B}(\bar{x}_{m.cb.}-\bar{x}_{m.c..})^2$	$MC(B-1)$	$\sigma^2+F\sigma_{BT}^2+TF\sigma_B^2$
	BioRep× TechRep (Condition × Mixture) (Subplot error)	random	$F\sum_{m=1}^{M}\sum_{t=1}^{T}\sum_{c=1}^{C}\sum_{b=1}^{B}(\bar{x}_{mtcb.}-\bar{x}_{mtc..}-\bar{x}_{m.cb.}+\bar{x}_{m.c..})^2$	$MC(B-1)$ $(T-1)$	$\sigma^2+F\sigma_{BT}^2$
Sub-subplot	Feature	fixed	$MTCB\sum_{f=1}^{F}(\bar{x}_{....f}-\bar{x}_{....})^2$	$F-1$	$\sigma^2+\frac{MTCB\sum_f F_f^2}{F-1}$
	Sub-subplot error	random	SS_{Error}	$(F-1)$ $(MTCB-1)$	σ^2
	Total		SS_{Total}	$MTCBF-1$	

Supplementary Figure S3.2: Analysis of variance (ANOVA) table for the model in Supplementary Figure S3.1 in a balanced design The sums of squares for the whole plot (in blue) and the subplot (in orange) do not depend on the observed values of log_2-intensities x_{mtcbf}, but only on the averages $\bar{x}_{mtcb.}$ over the features. Thus, inference from the full model can be performed in two steps, by first calculating the summaries, and next performing hypothesis testing for the subplot.

After protein summarization, the ANOVA table in Supplementary Figure S3.2 is reduced as follows.

	Source of variation	Effect	Sum of Squares	Degrees of freedom	Expected Mean Square
Whole plot	Mixture	random	$TCB\sum_{m=1}^{M}(\bar{y}_{m...}-\bar{y}_{....})^2$	$M-1$	$\sigma^2+T\sigma_B^2+CB\sigma_T^2+TCB\sigma_M^2$
	TechRep(Mixture) (Whole plot error)	random	$CB\sum_{m=1}^{M}\sum_{t=1}^{T}(\bar{y}_{mt..}-\bar{y}_{m...})^2$	$M(T-1)$	$\sigma^2+CB\sigma_T^2$
Subplot	Condition	fixed	$MTB\sum_{c=1}^{C}(\bar{y}_{..c.}-\bar{y}_{....})^2$	$C-1$	$\sigma^2+T\sigma_B^2+B\sigma_{CT}^2+TB\sigma_{CM}^2+\frac{MTB\sum_j C_j^2}{C-1}$
	Condition × Mixture	random	$TB\sum_{c=1}^{C}\sum_{m=1}^{M}(\bar{y}_{m.c.}-\bar{y}_{m...}-\bar{y}_{..c.}+\bar{y}_{....})^2$	$(C-1)(M-1)$	$\sigma^2+T\sigma_B^2+B\sigma_{CT}^2+TB\sigma_{CM}^2$
	Condition × TechRep(Mixture)	random	$B\sum_{m=1}^{M}\sum_{t=1}^{T}\sum_{c=1}^{C}(\bar{y}_{mtc.}-\bar{y}_{mt..}-\bar{y}_{m.c.}+\bar{y}_{m...})^2$	$M(C-1)$ $(T-1)$	$\sigma^2+B\sigma_{CT}^2$
	BioRep(Condition × Mixture)	random	$T\sum_{m=1}^{M}\sum_{c=1}^{C}\sum_{b=1}^{B}(\bar{y}_{m.cb}-\bar{y}_{m.c.})^2$	$MC(B-1)$	$\sigma^2+T\sigma_B^2$
	BioRep × TechRep (Condition × Mixture) (Subplot error)	random	$\sum_{m=1}^{M}\sum_{t=1}^{T}\sum_{c=1}^{C}\sum_{b=1}^{B}(\bar{y}_{mtcb}-\bar{y}_{mtc.}-\bar{y}_{m.cb}+\bar{y}_{m.c.})^2$	$MC(B-1)$ $(T-1)$	σ^2
	Total		SS_{Total}	$MTCB-1$	

Supplementary Figure S3.3: Reduced ANOVA table in Supplementary Figure S3.2 after protein summarization, in a balanced group comparison design with biological variation, multiple mixtures, and multiple technical replicates.

This model is further simplified by *MSstatsTMT* for different experimental designs, as shown below.

3.2 MSstatsTMT: group comparison designs with biological variation, multiple mixtures, and multiple technical replicates

Our prior empirical results (Supplementary Section S4) indicate that the variation due to the interaction $Condition \times TechRep(Mixture)_{ct(m)}$ (σ^2_{CT}) is relatively small . Therefore, *MSstatsTMT* pools $Condition \times TechRep(Mixture)_{ct(m)}$ with the error.

Further, the empirical results indicate that the variation due to the interaction $Condition \times Mixture_{cm}$ (σ^2_{CM}) is also relatively small. Therefore, to avoid overfitting, *MSstatsTMT* combines $Condition \times Mixture_{cm}$ with $BioRep(Mixture \times Condition)_{b(mc)}$ into a new term $Subject_{mcb}$. The term $Subject$ represents biological replicates, while using the convention that each biological replicate has a unique identifier across mixtures and conditions.

The resulting model is

$$Y_{mtcb} = \mu + Mixture_m + TechRep(Mixture)_{t(m)} + Condition_c + Subject_{mcb} + \varepsilon_{mtcb} \qquad (1)$$

$$\text{where } Mixture_m \overset{iid}{\sim} N(0, \sigma^2_M),\ TechRep(Mixture)_{t(m)} \overset{iid}{\sim} N(0, \sigma^2_T),\ \sum_{c=1}^{C} Condition_c = 0,$$

$$Subject_{mcb} \overset{iid}{\sim} N(0, \sigma^2_S),\ \varepsilon_{mtcb} \overset{iid}{\sim} N(0, \sigma^2)$$

The corresponding ANOVA table shown in Supplementary Figure S3.4. It is used by *MSstatsTMT* for designs with biological variation, multiple mixtures, and multiple technical replicates.

	Source of variation	Effect	Sum of Squares	Degrees of freedom	Expected Mean Square
Whole plot	Mixture	random	$TCB \sum_{m=1}^{M} (\bar{y}_{m...} - \bar{y}_{....})^2$	$M - 1$	$\sigma^2 + T\sigma^2_S + CB\sigma^2_T + TCB\sigma^2_M$
	TechRep(Mixture)	random	$CB \sum_{m=1}^{M} \sum_{t=1}^{T} (\bar{y}_{mt..} - \bar{y}_{m...})^2$	$M(T - 1)$	$\sigma^2 + CB\sigma^2_T$
Subplot	Condition	fixed	$MTB \sum_{c=1}^{C} (\bar{y}_{..c.} - \bar{y}_{....})^2$	$C - 1$	$\sigma^2 + T\sigma^2_S + \frac{MTB \sum_j C_j^2}{C - 1}$
	Subject	random	$T \sum_{m=1}^{M} \sum_{c=1}^{C} \sum_{b=1}^{B} (\bar{y}_{m.cb} - \bar{y}_{m...} - \bar{y}_{..c.} + \bar{y}_{....})^2$	$MCB - M - C + 1$	$\sigma^2 + T\sigma^2_S$
	Error	random	$\sum_{m=1}^{M} \sum_{t=1}^{T} \sum_{c=1}^{C} \sum_{b=1}^{B} (\bar{y}_{mtcb} - \bar{y}_{m.cb} - \bar{y}_{mt..} + \bar{y}_{m...})^2$	$M(TCB - CB - T + 1)$	σ^2
	Total		SS_{Total}	$MTCB - 1$	

Supplementary Figure S3.4: Analysis of variance table for the model in Supplementary Equation S1, used by *MSstatsTMT* in balanced group comparison designs with biological variation, multiple mixtures, and multiple technical replicates The term $Subject$ represents biological replicates, while using the convention that each biological replicate has a unique identifier across mixtures and conditions

3.3 MSstatsTMT: group comparison designs with biological variation, multiple mixtures, and single technical replicate

When the experiment does not have technical replicates, the terms involving technical replicates, i.e., $TechRep(Mixture)$ and ε, are not estimable and are therefore omitted. The model in Equation (3) of main text is simplified to

$$Y_{mcb} = \mu + Mixture_m + Condition_c + \varepsilon_{mcb} \quad (2)$$
$$\text{where } Mixture_m \overset{iid}{\sim} N(0, \sigma_M^2),\ \sum_{c=1}^{C} Condition = 0,\ \varepsilon_{mcb} \overset{iid}{\sim} N(0, \sigma^2)$$

The term ε combines the biological variation and the technical variation that is not explained by $Mixture$. Since the terms $TechRep(Mixture)_{t(m)}$ and ϵ_{mtcb} in Supplementary Figure S3.4 are not estimable, the term $Subject$ expressing the biological variation becomes the subplot error. The variance components in the general linear model in Supplementary Figure S3.4 in a balanced case are then reduced to Supplementary Figure S3.5.

	Source of variation	Effect	Sum of Squares	Degrees of freedom	Expected Mean Square
Whole plot	Mixture	random	$CB\sum_{m=1}^{M}(\bar{y}_{m..} - \bar{y}_{...})^2$	$M-1$	$\sigma^2 + CB\sigma_M^2$
Subplot	Condition	fixed	$MB\sum_{c=1}^{C}(\bar{y}_{.c.} - \bar{y}_{...})^2$	$C-1$	$\sigma^2 + \frac{MB\sum_j C_j^2}{C-1}$
	Error	random	$\sum_{m=1}^{M}\sum_{c=1}^{C}\sum_{b=1}^{B}(\bar{y}_{mcb} - \bar{y}_{.c.} - \bar{y}_{m..} + \bar{y}_{...})^2$	$MCB - M - C + 1$	σ^2
	Total		SS_{Total}	$MCB-1$	

Supplementary Figure S3.5: Analysis of variance table for the model in Supplementary Equation S2, used by *MSstatsTMT* in balanced group comparison designs with biological variation, multiple mixtures, and single technical replicate This is the final model used by *MSstatsTMT* for designs with biological variation, multiple mixtures, and single technical replicate.

3.4 MSstatsTMT: group comparison designs with biological variation, single mixture, and multiple technical replicates

When the experimental design only includes a single mixture, but multiple technical replicate runs, the term $Mixture$ is not estimable. The model in Equation (3) of main text becomes

$$Y_{tcb} = \mu + TechRep_t + Condition_c + Subject_{cb} + \varepsilon_{tcb} \tag{3}$$

$$\text{where } TechRep_t \overset{iid}{\sim} N(0, \sigma_T^2),\ \sum_{c=1}^{C} Condition = 0,\ Subject_{cb} \overset{iid}{\sim} N(0, \sigma_S^2),\ \varepsilon_{tcb} \overset{iid}{\sim} N(0, \sigma^2)$$

The term ε represents the technical variation that is not explained by $TechRep$. Since the term $Mixture_m$ in Supplementary Figure S3.4 is not estimable, the variance components in the general linear model in Supplementary Figure S3.4 in a balanced case are reduced to Supplementary Figure S3.6.

	Source of variation	Effect	Sum of Squares	Degrees of freedom	Expected Mean Square
Whole plot	TechRep	random	$CB\sum_{t=1}^{T}(\bar{y}_{t..} - \bar{y}_{...})^2$	$T-1$	$\sigma^2 + CB\sigma_T^2$
Subplot	Condition	fixed	$TB\sum_{c=1}^{C}(\bar{y}_{.c.} - \bar{y}_{...})^2$	$C-1$	$\sigma^2 + T\sigma_S^2 + \frac{TB\sum_j C_j^2}{C-1}$
	Subject	random	$T\sum_{c=1}^{C}\sum_{b=1}^{B}(\bar{y}_{.cb} - \bar{y}_{.c.})^2$	$CB-C$	$\sigma^2 + T\sigma_S^2$
	Error	random	$\sum_{t=1}^{T}\sum_{c=1}^{C}\sum_{b=1}^{B}(\bar{y}_{tcb} - \bar{y}_{.cb} - \bar{y}_{t..} + \bar{y}_{...})^2$	$TCB-CB-T+1$	σ^2
	Total		SS_{Total}	$TCB-1$	

Supplementary Figure S3.6: Analysis of variance table for the model in Supplementary Equation S3, used by *MSstatsTMT* in balanced group comparison designs with biological variation, single mixtures, and multiple technical replicates

3.5 MSstatsTMT: group comparison designs with biological variation, single mixture, and single technical replicate

When the experiment only has one mixture profiled in a single technical replicate MS run, the terms $TechRep(Mixture)$, $Mixture$ and ε are not estimable. The model in Equation (3) of main text is reduced to

$$Y_{cb} = \mu + Condition_c + \varepsilon_{cb} \tag{4}$$
$$\text{where } \sum_{c=1}^{C} Condition = 0,\ \varepsilon_{cb} \overset{iid}{\sim} N(0, \sigma^2)$$

The term ε combines all the biological and technical variation. The variance components in the general linear model in Supplementary Figure S3.4 in a balanced case are reduced to Supplementary Figure S3.7.

Source of variation	Effect	Sum of Squares	Degrees of freedom	Expected Mean Square
Condition	fixed	$B\sum_{c=1}^{C}(\bar{y}_{c.} - \bar{y}_{..})^2$	$C - 1$	$\sigma^2 + \frac{B\sum_j C_j^2}{C-1}$
Error	random	$\sum_{c=1}^{C}\sum_{b=1}^{B}(\bar{y}_{cb} - \bar{y}_{c.})^2$	$CB - C$	σ^2
Total		SS_{Total}	$CB - 1$	

Supplementary Figure S3.7: Analysis of variance table for the model in Supplementary Equation S4, used by *MSstatsTMT* in balanced group comparison designs with biological variation, single mixture, and single technical replicate

3.6 MSstatsTMT: group comparison designs without biological variation (controlled mixtures)

Controlled mixtures with spiked-in proteins are mainly used for purposes of model development and benchmarking. They do not contain biological variation and the term $Subject_{bmc}$ in Supplementary Figure S3.4 is not estimable. The model in *MSstatsTMT* was adapted to these cases by removing the term $Subject$, as follows.

$$Y_{mtcb} = \mu + Mixture_m + TechRep(Mixture)_{t(m)} + Condition_c + \varepsilon_{mtcb} \tag{5}$$
$$\text{where } Mixture_m \overset{iid}{\sim} N(0, \sigma_M^2),\ TechRep(Mixture)_{t(m)} \overset{iid}{\sim} N(0, \sigma_T^2),$$
$$\sum_{c=1}^{C} Condition = 0,\ \varepsilon_{mtcb} \overset{iid}{\sim} N(0, \sigma^2)$$

Supplementary Figure S3.8 shows the Analysis of Variance (ANOVA) decomposition for this model in the case of a balanced design.

	Source of variation	Effect	Sum of Squares	Degrees of freedom	Expected Mean Square
Whole plot	Mixture	random	$TCB\sum_{m=1}^{M}(\bar{y}_{m...} - \bar{y}_{....})^2$	$M-1$	$\sigma^2 + CB\sigma_T^2 + TCB\sigma_M^2$
	TechRep(Mixture)	random	$CB\sum_{m=1}^{M}\sum_{t=1}^{T}(\bar{y}_{mt..} - \bar{y}_{m...})^2$	$M(T-1)$	$\sigma^2 + CB\sigma_T^2$
Subplot	Condition	fixed	$MTB\sum_{c=1}^{C}(\bar{y}_{..c.} - \bar{y}_{....})^2$	$C-1$	$\sigma^2 + \frac{MTB\sum_j C_j^2}{C-1}$
	Error	random	$\sum_{m=1}^{M}\sum_{t=1}^{T}\sum_{c=1}^{C}\sum_{b=1}^{B}(\bar{y}_{mtcb} - \bar{y}_{mt..} - \bar{y}_{..c.} + \bar{y}_{....})^2$	$MTCB - MT - C + 1$	σ^2
	Total		SS_{Total}	$MTCB-1$	

Supplementary Figure S3.8: Analysis of variance table for the model in Supplementary Equation S5, used by *MSstatsTMT* in balanced group comparison designs with biological variation, single mixture, and single technical replicate

When the experiment does not have technical replicates, *MSstatsTMT* fits the same Equation (2). Similarly, if the experiment only has one mixture profiled in a single technical replicate MS run, Equation (4) is used.

3.7 MSstatsTMT: Details of parameter estimation and testing for differential abundance

Pairwise comparisons between conditions Model-based testing for differentially abundant proteins between pairs of conditions is carried out through a contrast of the condition means. Denote $\beta = (\beta_1, \beta_2, \ldots, \beta_C)$ the parameters associated with the terms *Condition* in Supplementary Equation S1-5, and $l = (l_1, l_2, \ldots, l_C)$ a vector of coefficients where $\sum l_c = 0$. A contrast is defined as $l^T\beta = \sum_c l_c\beta_c$. For example, a pairwise comparison testing proteins for differential abundance between Condition 1 and Condition 2 can be expressed as a contrast with $l = (1, -1, 0, \ldots, 0)$. We are interested in the null hypothesis $l^T\beta = \sum l_c\beta_c = 0$.

We estimate the parameters of the model using restricted maximum likelihood [7, 8, 9] to obtain $\hat{\beta}$, the contrast $l^T\hat{\beta} = \sum_c l_c\hat{\beta}_c$, and the corresponding t-statistic [10]

$$t = \frac{l^T\hat{\beta}}{\sqrt{ls^2\hat{V}l^T}} \tag{6}$$

Here s^2 is the estimate of σ^2 in Supplementary Equation S1, and $\hat{V}$ is the unscaled variance-covariance matrix of $\hat{\beta}$, such that $s^2\hat{V}$ is the variance-covariance matrix of $\hat{\beta}$, and $\sqrt{ls^2\hat{V}l^T}$ is the standard error of the contrast. The estimates s^2 and $\hat{V}$ are obtained by restricted maximum likelihood. The matrix $\hat{V}$ is a function of estimates $\hat{\sigma}_M^2$ for the random effect of mixtures, $\hat{\sigma}_T^2$ for the random effect of technical replicates, and $\hat{\sigma}_S^2$ for the random effect of subject. Therefore, the standard error of the contrast takes into account both technical variance σ_M^2, σ_T^2, σ^2 and biological variance σ_S^2.

In general and unbalanced designs, the degrees of freedom of the t-statistic in Supplementary Equation S6 are derived by Satterthwaite approximation [11]

$$df = \frac{2(s^2l\hat{V}l^T)^2}{[\text{VAR}(s^2l\hat{V}l^T)]}. \tag{7}$$

The calculation of $[\text{VAR}(s^2l\hat{V}l^T)]$ is described in [12, 10]. In unbalanced design, different contrasts of a same protein may have different degrees of freedom.

Empirical Bayes variance moderation When the number of biological replicates in each condition is small, we adopt Empirical Bayes moderation from *limma* [13]. Briefly, we assume that the estimate of the error variance σ^2 in Supplementary Equation S1 s^2 follows a scaled chi-square distribution with ν degrees of freedom

$$s^2|\sigma^2 \sim \frac{\sigma^2}{\nu}\chi^2_\nu, \tag{8}$$

The variance σ^2 of each protein is in turn assumed to follow a scaled inverse chi-square prior distribution with prior degrees of freedom d_0 and prior variance s_0^2

$$\frac{1}{\sigma^2} \sim \frac{1}{d_0 s_0^2}\chi^2_{d_0}. \tag{9}$$

The degree of freedom ν is estimated as implemented in the R package lmerTest [12]. The parameters d_0 and s_0^2 are estimated from the distribution of the observed s^2 of all the proteins using an Empirical Bayes approach as implemented in the R package *limma* [14]. Finally, the posterior variance estimate is incorporated into the residual variance of each protein

$$\tilde{s}^2 = \frac{s_0^2 d_0 + s^2\nu}{d_0 + \nu}. \tag{10}$$

Then, the moderated t-statistic for the contrast l becomes

$$\tilde{t} = \frac{l^T\hat{\beta}}{\sqrt{l\tilde{s}^2\hat{V}l^T}}, \tag{11}$$

with $df + d_0$ degrees of freedom.

Section 4: Empirical details of model development in MSstatsTMT

4.1 Mouse-3mix-unbalanced

We used the Mouse-3mix-unbalanced experiment as a representative case, to characterize the relative importance of the sources of variation in the linear mixed effects model in Supplementary Figure S3.3. The effects $TechRep(Mixture)_{ct(m)}$, $Condition \times TechRep(Mixture)_{ct(m)}$ and the subplot error are not estimable since Mouse-3mix-unbalanced has single technical replicate MS run per mixture. The magnitude of the variance components is summarized in Supplementary Figure S4.1. The variances of $Condition \times Mixture$ (median 11.8%) were negligibly small, compared to $Mixture$ (median 36.3%) and the subplot error (median 32.4%). Therefore, to avoid overfitting, *MSstatsTMT* removes the interaction $Condition \times Mixture$ from the model in Supplementary Figure S3.3.

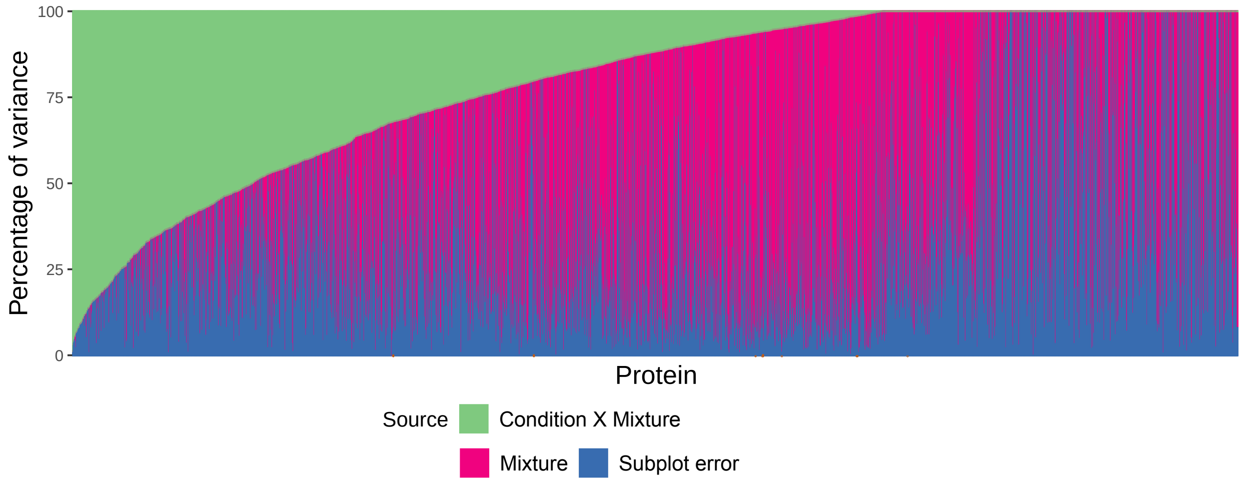

Supplementary Figure S4.1: Variance components in the general linear mixed effects model in Equation (2) of main text, fit to the normalized protein-level summaries of Mouse-3mix-unbalanced experiment. The x-axis represents 3,471 proteins to fit the model. For each protein, the colors indicate the percentage of each variance component in the total variation. The median percentage of $Condition \times Mixture$, $Mixture$ and subplot error over all the proteins is 11.8%, 36.3% and 32.4% respectively. The largest components of variation are the subplot error and the $Mixture$, and the variance components of $Condition \times Mixture$ are negligibly small.

4.2 SpikeIn-5mix-MS3

We used the SpikeIn-5mix-MS3 experiment as a representative case, to characterize the relative importance of the sources of variation in the general linear mixed effects model in Supplementary Figure S3.3. The $BioRep$ effect is not estimable since SpikeIn-5mix-MS3 does not have biological variation. The magnitude of the variance components is summarized in Supplementary Figure S4.2. The variances of $Mixture$ and of $TechRep(Mixture)$ were small, the median percentage of which is 0% and 23.1% respectively, due to the effect of the normalization with respect to the reference channel. The subplot error (median 63.3%) was relatively large compared to $Condition \times Mixture$ and $Condition \times TechRep(Mixture)$ interactions (median 3.22%). Therefore, to avoid overfitting, *MSstatsTMT* removed these interactions from the model in Supplementary Figure S3.3.

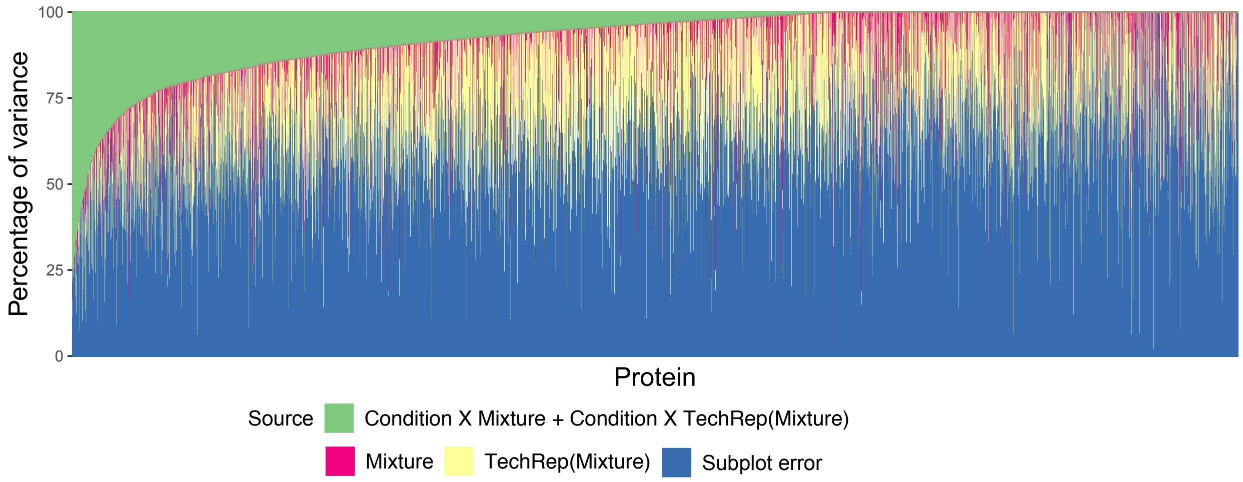

Supplementary Figure S4.2: Variance components in the general linear mixed effects model in Supplementary Equation S5, fit to the normalized protein-level summaries of SpikeIn-5mix-MS3 experiment The x-axis represents 4,313 proteins which have enough data to fit the model. For each protein, the colors indicate the percentage of each variance component in the total variation. The median percentage of interactions $Condition \times Mixture + Condition \times TechRep(Mixture)$, $Mixture$, $TechRep(Mixture)$ and subplot error over all the proteins is 3.22%, 0%, 23.1%, 63.3% respectively. The largest component of variation comes from subplot error (i.e., differences in the individual protein profiles). The variance components of $Mixture$, $Condition \times Mixture + Condition \times TechRep(Mixture)$ are negligibly small.

Section 5: Evaluation of performance : SpikeIn-5mix-MS3 and SpikeIn-5mix-MS2 experiment

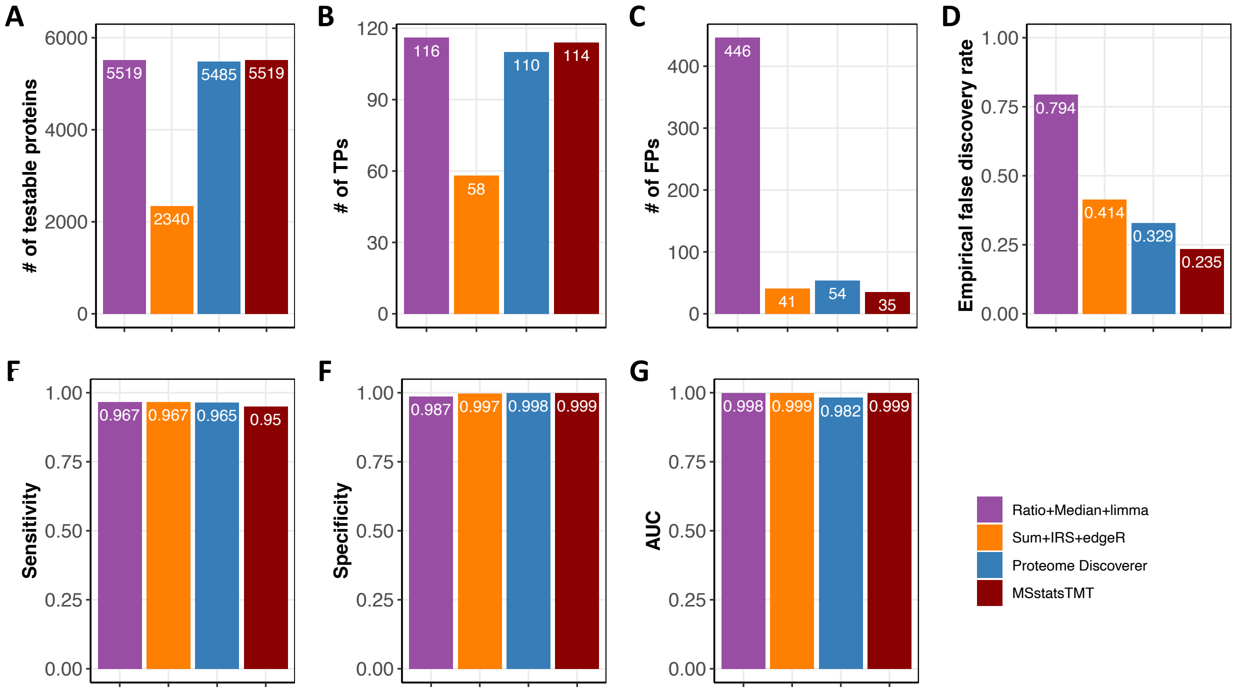

Supplementary Figure S5.1: Detection of differentially abundant proteins in all pairs of conditions in SpikeIn-5mix-MS2 (FDR cutoff of 0.05) Colors represent statistical modeling and inference methods in Table 1. (A) Number of testable proteins. (B) Number of true positive differentially abundant proteins. (C) Number of false positive differentially abundant proteins. (D) Empirical false discovery rate. (E) Sensitivity of correctly detecting the spiked-in proteins. (F) Specificity of correctly detecting the background proteins. (G) Area under ROC curve (AUC).

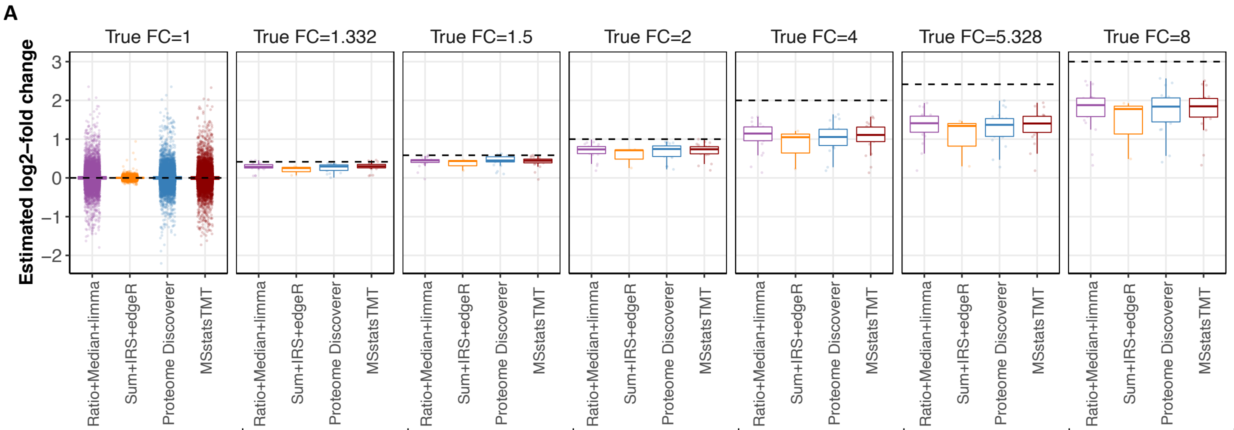

Median	0	0	-0.001	-0.003	0.285	0.255	0.294	0.3	0.452	0.436	0.449	0.443	0.736	0.714	0.745	0.739	1.145	1.051	1.056	1.109	1.411	1.341	1.37	1.404	1.878	1.777	1.84	1.848
IQR	0.07	0.029	0.07	0.072	0.087	0.112	0.144	0.09	0.071	0.134	0.133	0.096	0.183	0.235	0.274	0.184	0.356	0.489	0.418	0.375	0.414	0.584	0.46	0.416	0.476	0.718	0.619	0.481
# of proteins	4,791	1,926	4,719	4,791	21	3	21	21	21	3	21	21	21	3	21	21	21	3	21	21	21	3	21	21	21	3	21	21

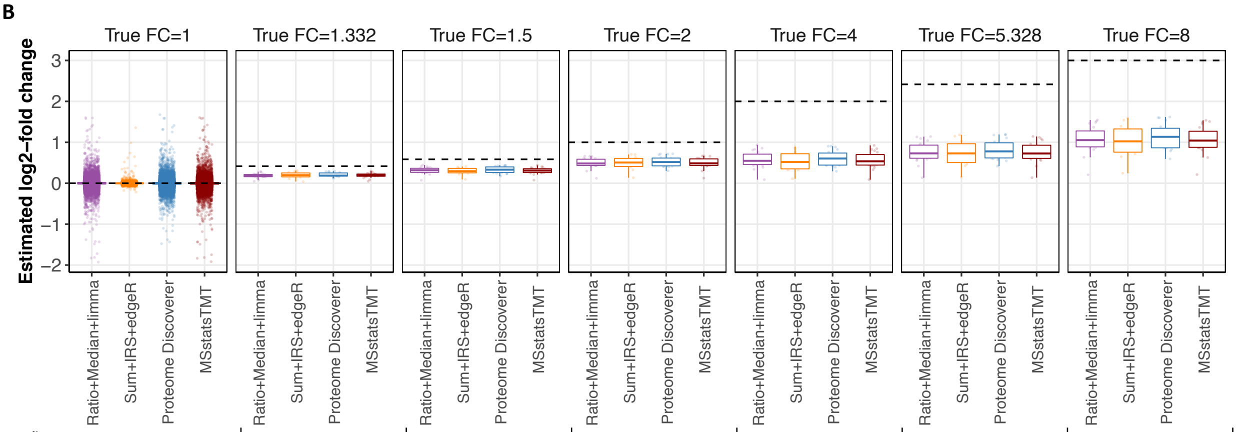

Median	0	-0.001	-0.003	-0.003	0.184	0.197	0.185	0.191	0.319	0.295	0.33	0.309	0.483	0.506	0.517	0.486	0.546	0.518	0.604	0.536	0.73	0.729	0.778	0.727	1.053	1.023	1.133	1.042
IQR	0.038	0.017	0.04	0.04	0.054	0.097	0.077	0.055	0.099	0.108	0.139	0.094	0.151	0.197	0.19	0.161	0.254	0.37	0.295	0.257	0.321	0.461	0.371	0.321	0.389	0.569	0.476	0.393
# of proteins	5,499	2,330	5,466	5,499	20	10	19	20	20	10	19	20	20	10	19	20	20	10	19	20	20	10	19	20	20	10	19	20

Supplementary Figure S5.2: Distribution of estimated log_2 fold change (FC) in the SpikeIn-5mix-MS3 and SpikeIn-5mix-MS2 experiment. Each panel is the true fold change of the proteins between pairs of conditions, true fold change = 1 corresponds to the background proteins. X-axis: workflows in Table 1. Horizontal line indicates the true log_2 fold change. Tables below the boxplots show median of the estimated log_2 fold changes and interquartile range (IQR, distance between 75th and 25th percentile) over all the proteins. '# of proteins' is the number of testable proteins. (A) Distribution of estimated log_2 fold change (FC) in the SpikeIn-5mix-MS3. All four methods underestimated the fold change of the spiked proteins (FC $>$ 1) to some extent. This may be due to the low abundance ($<$1%) of spiked-in peptides as compared to the background peptides (approximately 14 ng as compared to 2 ug for the UPS1 and SILAC-HeLa peptides, respectively). (B) Distribution of estimated log_2 fold change (FC) in the SpikeIn-5mix-MS2. The estimates by the four methods are similar and all underestimate the true fold changes of spiked-in proteins (FC $>$ 1). In particular, the underestimation of fold changes (also called ratio compression) is worse in the MS2-based quantification than that in the MS3-based quantification.The results confirm that MS3-based quantification can reduce the ratio compression.

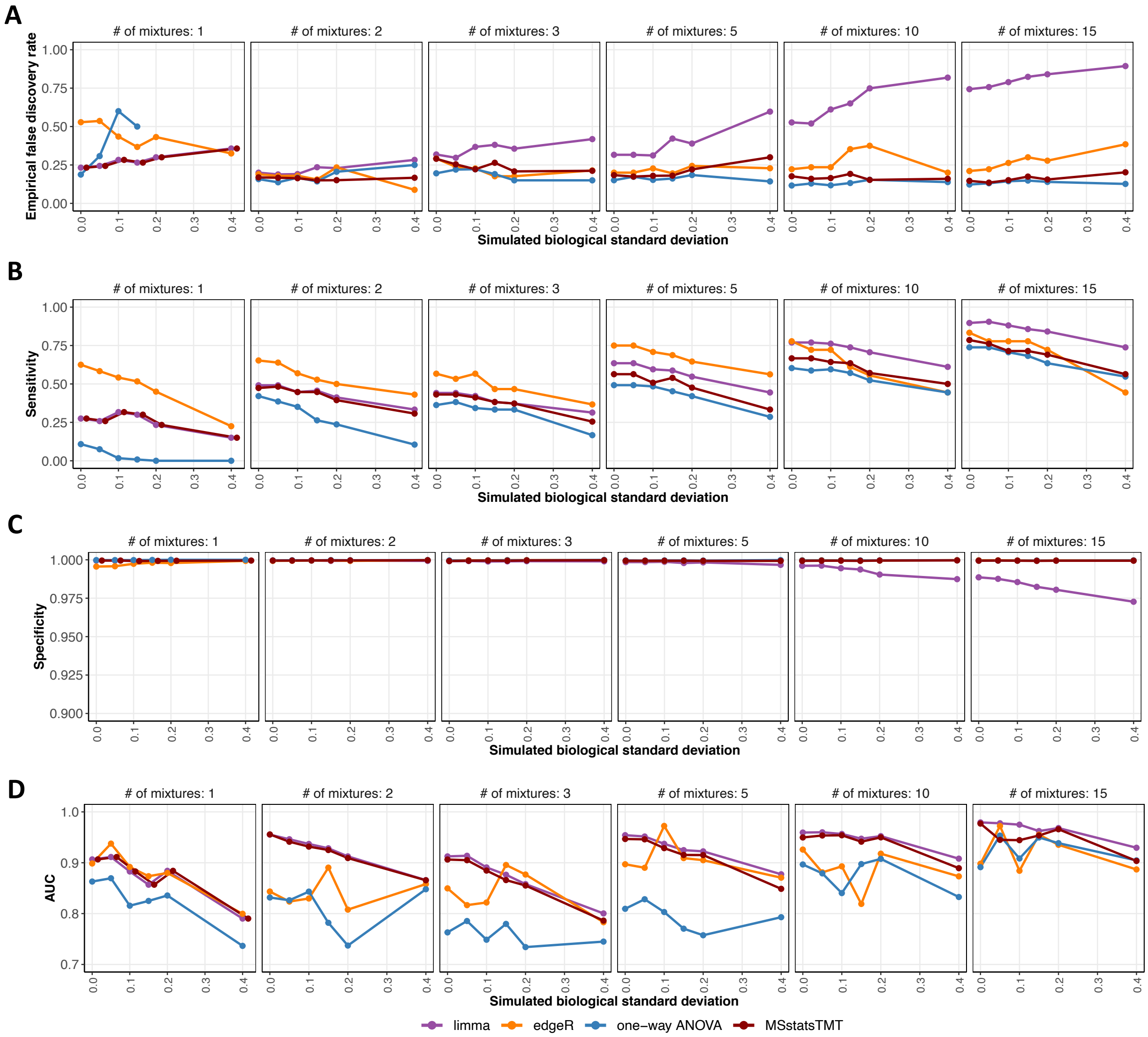

Supplementary Figure S5.3: Detection of differentially abundant proteins in all pairs of conditions in SpikeIn-15mix-MS3-Sim (FDR cutoff of 0.05). SpikeIn-15mix-MS3-Sim experiment simulated 15 biological mixtures and no technical replicates. Panels in the figure represent randomly selected subsets of 1, 2, 3, 5, 10 mixtures, and 15 mixtures. Colors represent statistical modeling and inference methods in Table 1. X-axis: simulated biological standard deviation (standard deviation = 0 corresponds to the original controlled mixtures SpikeIn-5mix-MS3). (A) Empirical false discovery rate. (B) Sensitivity of correctly detecting the spiked-in proteins. (C) Specificity of correctly detecting the background proteins. (D) Area under ROC curve (AUC).

A

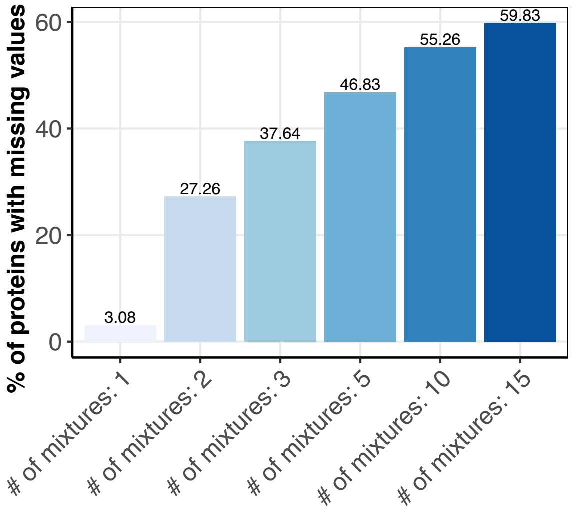

B

	# of mixtures: 1	# of mixtures: 2	# of mixtures: 3	# of mixtures: 5	# of mixtures: 10	# of mixtures: 15
limma	3,331	3,781	4,008	4,265	4,633	4,812
edgeR	3,228	2,748	2,496	2,267	2,073	1,933
One-way ANOVA	3,320	3,771	3,989	4,248	4,626	4,798
MSstatsTMT	3,331	3,781	4,008	4,265	4,633	4,812

Supplementary Figure S5.4: Summary of SpikedIn-15mix-MS3-Sim experiment. SpikeIn-15mix-MS3-Sim experiment simulated a total of 15 runs, with 15 biological mixtures (30 biological replicates per condition), and no technical replicates. Panels in the figure represent randomly selected subsets of 1, 2, 3, 5, 10 mixtures, and 15 mixtures. (A) Percentage of proteins with at least one missing value. (B) Number of testable proteins.

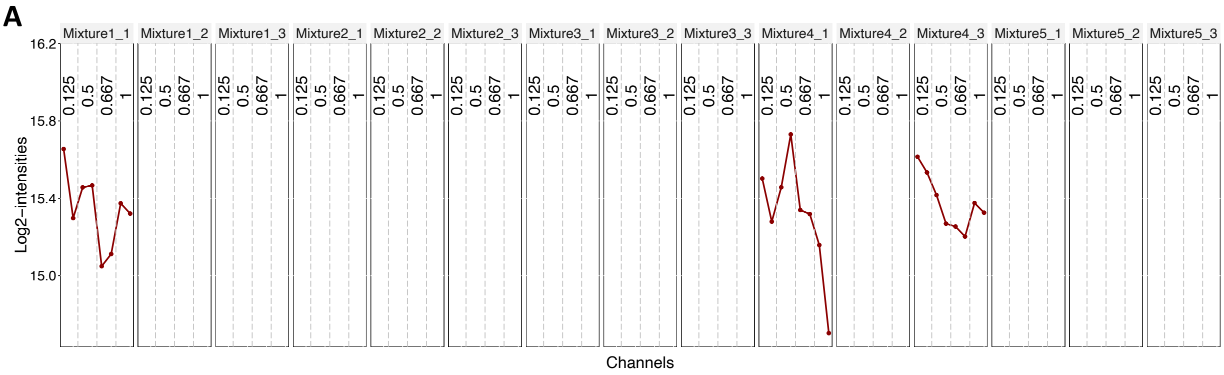

B **MSstatsTMT**: $Y_{mcb} = \mu + Mixture_m + Condition_c + \varepsilon_{mcb}$

$$Mixture_m \sim N(0, \sigma_M^2) \quad \sum_c Condition_c = 0 \quad \varepsilon_{mtcb} \sim N(0, \sigma^2)$$

**Background protein O00399**	Variance estimates	Log2 FC	SE	DF	P-value	Adjusted p-value
Comparison: **0.125-0.667**	$\hat{\sigma}_M^2 = 0.0000$ $\hat{\sigma}^2 = 0.0322$	0.2679	0.1194	26.29	0.0335	0.1752

C **limma**: $Y_{mcb} = \mu + Mixture_m + Condition_c + \varepsilon_{mcb}$

$$\sum_m Mixture_m = 0 \quad \sum_c Condition_c = 0 \quad \varepsilon_{mtcb} \sim N(0, \sigma^2)$$

**Background protein O00399**	Variance estimates	Log2 FC	SE	DF	P-value	Adjusted p-value
Comparison: **0.125-0.667**	$\hat{\sigma}^2 = 0.0348$	0.2679	0.0703	24.41	0.0008	0.0391

D **One-way ANOVA**: $Y_{mtcb} = \mu + Condition_c + \varepsilon_{mtcb}$

$$\sum_c Condition_c = 0 \quad \varepsilon_{mtcb} \sim N(0, \sigma^2)$$

**Background protein O00399**	Variance estimates	Log2 FC	SE	DF	P-value	Adjusted p-value
Comparison: **0.125-0.667**	$\hat{\sigma}^2 = 0.0321$	0.2679	0.1035	20	0.0176	0.9994

E **edgeR**: $2^{Y_{mtcb}} \sim NB(\mu_{mtb} * Condition_c, \phi)$ $\quad \phi$ is the dispersion

**Background protein O00399**	Log2 FC	SE	DF	P-value	Adjusted p-value
Comparison: **0.125-0.667**	NA	NA	NA	NA	NA

Supplementary Figure S5.5: Background protein O00399 (Dynactin subunit 6, DCTN6_human) from SpikeIn-5mix-3TechRep-MS3-Sim. The experiment had a total of 15 runs, with 15 biological mixtures (30 biological replicates per condition) and no technical replicates. The simulated biological standard deviation was set to 0.2. The protein was only observed in three runs. (A) Normalized log_2 protein-level summaries by *MSstatsTMT*. X-axis: TMT channels. Y-axis: log_2 intensity. Each panel represents one MS run. Labels inside each panel are conditions of the corresponding channel and mixture. (B) *MSstatsTMT*: model and model-based inference comparing condition 0.125 and 0.667. Since this is a background protein, the true log_2 fold change is 0. The protein was not found differentially abundant. (C) *limma*: model and model-based inference comparing condition 0.125 and 0.667. *limma* did not separate the biological and the technical variation. This inflated the degrees of freedom associated with the comparison, and resulted in a false positive differentially abundant proteins. (D) *One-way ANOVA* implemented in *Proteome Discoverer*: same conclusion as in (C). (E) *EdgeR*: model and model-based inference comparing condition 0.125 and 0.667. *EdgeR* was unable to estimate the standard error of the estimated fold change, but reported the protein as differentially abundant.

References

[1] Rappsilber, J., Mann, M., and Ishihama, Y. (2007). Protocol for micro-purification, enrichment, pre-fractionation and storage of peptides for proteomics using StageTips. *Nature protocols*, **2**, 1896.

[2] Paulo, J. A., McAllister, F. E., Everley, R. A., Beausoleil, S. A., Banks, A. S., and Gygi, S. P. (2015). Effects of MEK inhibitors GSK1120212 and PD0325901 in vivo using 10-plex quantitative proteomics and phosphoproteomics. *Proteomics*, **15**, 462–473.

[3] McAlister, G. C., Nusinow, D. P., Jedrychowski, M. P., Wuhr, M., Huttlin, E. L., Erickson, B. K., Rad, R., Haas, W., and Gygi, S. P. (2014). Multinotch ms3 enables accurate, sensitive, and multiplexed detection of differential expression across cancer cell line proteomes. *Analytical Chemistry*, **86**, 7150–7158.

[4] Paulo, J. A., O'Connell, J. D., and Gygi, S. P. (2016). A triple knockout (TKO) proteomics standard for diagnosing ion interference in isobaric labeling experiments. *Journal of the American Society for Mass Spectrometry*, **27**, 1620–1625.

[5] Djomehri, S. I., Gonzalez, M. E., da Veiga Leprevost, F., Tekula, S. R., Chang, H.-Y., White, M. J., Cimino-Mathews, A., Burman, B., Basrur, V., Argani, P., *et al.* (2020). Quantitative proteomic landscape of metaplastic breast carcinoma pathological subtypes and their relationship to triple-negative tumors. *Nature communications*, **11**, 1–15.

[6] Plubell, D. L., Wilmarth, P. A., Zhao, Y., Fenton, A. M., Minnier, J., Reddy, A. P., Klimek, J., Yang, X., David, L. L., and Pamir, N. (2017). Extended multiplexing of tandem mass tags (TMT) labeling reveals age and high fat diet specific proteome changes in mouse epididymal adipose tissue. *Molecular & Cellular Proteomics*, **16**, 873–890.

[7] Bates, D., Mächler, M., Bolker, B., and Walker, S. (2015). Fitting linear mixed-effects models using lme4. *Journal of Statistical Software*, **67**, 1–48.

[8] Patterson, H. D. and Thompson, R. (1971). Recovery of inter-block information when block sizes are unequal. *Biometrika*, **58**, 545–554.

[9] Harville, D. A. (1977). Maximum likelihood approaches to variance component estimation and to related problems. *Journal of the American Statistical Association*, **72**, 320–338.

[10] Wang, T. and Merkle, E. C. (2018). merderiv: Derivative computations for linear mixed effects models with application to robust standard errors. *Journal of Statistical Software, Code Snippets*, **87**, 1–16.

[11] Satterthwaite, F. E. (1946). An approximate distribution of estimates of variance components. *Biometrics bulletin*, **2**, 110–114.

[12] Kuznetsova, A., Brockhoff, P. B., and Christensen, R. H. B. (2017). lmertest package: tests in linear mixed effects models. *Journal of statistical software*, **82**.

[13] Smyth, G. (2004). Linear models and empirical bayes methods for assessing differential expression in microarray experiments. *Statistical Applications in Genetics and Molecular Biology*, **3**, Article 3.

[14] Ritchie, M. E., Phipson, B., Wu, D., Hu, Y., Law, C. W., Shi, W., and Smyth, G. K. (2015). limma powers differential expression analyses for RNA-sequencing and microarray studies. *Nucleic Acids Research*, **43**, e47–e47.
